# Animal movement ecology in India: insights from 2011–2021 and prospective for the future

**DOI:** 10.7717/peerj.14401

**Published:** 2022-12-13

**Authors:** Harish Prakash, R. Suresh Kumar, Bibhuti Lahkar, Raman Sukumar, Abi T. Vanak, Maria Thaker

**Affiliations:** 1 Centre for Ecological Sciences, Indian Institute of Science, Bengaluru, Karnataka, India; 2 Department of Endangered Species Management, Wildlife Institute of India, Dehradun, Uttarakhand, India; 3 Aaranyak, Guwahati, Assam, India; 4 Ashoka Trust for Research in Ecology and the Environment, Bengaluru, Karnataka, India; 5 School of Life Sciences, University of KwaZulu-Natal, Durban, South Africa

**Keywords:** Movement ecology, Tracking, Telemetry, Home-range, Protected areas, Anthropogenic, Climate change, Wildlife conflict, Conservation, India

## Abstract

The field of animal movement ecology has advanced by leaps and bounds in the past few decades with the advent of sophisticated technology, advanced analytical tools, and multiple frameworks and paradigms to address key ecological problems. Unlike the longer history and faster growth of the field in North America, Europe, and Africa, movement ecology in Asia has only recently been gaining momentum. Here, we provide a review of the field from studies based in India over the last 11 years (2011–2021) curated from the database, Scopus, and search engine, Google Scholar. We identify current directions in the research objectives, taxa studied, tracking technology and the biogeographic regions in which animals were tracked, considering the years since the last systematic review of movement ecology research in the country. As an indication of the growing interest in this field, there has been a rapid increase in the number of publications over the last decade. Class *Mammalia* continues to dominate the taxa tracked, with tiger and leopard being the most common species studied across publications. Invertebrates and other small and medium-sized animals, as well as aquatic animals, in comparison, are understudied and remain among the important target taxa for tracking in future studies. As in the previous three decades, researchers have focussed on characterising home ranges and habitat use of animals. There is, however, a notable shift to examine the movement decision of animals in human-modified landscapes, although efforts to use movement ecology to understand impacts of climate change remain missing. Given the biogeographic and taxonomic diversity of India, and the fact that the interface between anthropogenic activity and wildlife interactions is increasing, we suggest ways in which the field of movement ecology can be expanded to facilitate ecological insights and conservation efforts. With the advancement of affordable technologies and the availability of analytical tools, the potential to expand the field of movement ecology, shift research foci, and gain new insights is now prime.

## Introduction

Animals travel a wide range of distances during their lifetime primarily in order to feed, find mates and seek refuge, among other critical behaviours (Bell, 1990). The terrestrial caribou *Rangifer tarandus*, for example, travels a total of ~4,000 km in a year (Joly et al., 2019) while the airborne arctic tern *Sterna paradisaea*, travels ~80,000 km during the same period (Egevang et al., 2010). In contrast to these long-distance migrants, the near stream-dwelling fire salamander *Salamandra Salamandra*, has a maximum annual displacement of only ~0.5 km (Schulte, Küsters & Steinfartz, 2007). Ecologists have, for decades, been interested in understanding the drivers of such travel distances (Holyoak et al., 2008). The movement ecology paradigm provides an explicit framework to understand movement patterns and drivers, considering the role of the abiotic environment, as well as the animal’s internal physiology, capacity to process information and navigate, and biomechanical capability to move (Nathan et al., 2008). This multi-faceted and unifying framework that enables the study of animal movement is complemented with recent technological developments of bio-loggers embedded with multiple sensors (Börger et al., 2020). Loggers not only track movement with GPS and accelerometery across space and time, but can also collect information on the animal’s external environment (*e.g*., ambient temperature, salinity, and light levels), and its internal physiological condition (*e.g*., body temperature, heart rate, and neurological state) (reviewed in Sherub et al., 2017). The latest low-cost miniature tags (~3 g in weight) also allow fine scale animal tracking at a global scale using satellites (Jetz et al., 2022). Thus, the integration of conceptual and analytical frameworks with current technological developments have been critical for gaining valuable insights into animal movement decisions of taxa inhabiting diverse environments across the globe.

Movement ecology has direct implications for understanding survival strategies of species, community structures and biodiversity (Jeltsch et al., 2013), and for generating conservation plans (Fraser et al., 2018). For example, movement data from the Carnaby’s cockatoo *Calyptorhynchus latirostris*, shows how travel distances for foraging are lower in contiguous habitats than in fragmented habitats, and this behaviour correlates with greater breeding success for the bird in contiguous habitats (Doherty & Driscoll, 2018). Movement of individuals can have larger ecological consequences beyond individual fitness or species distribution. The movement patterns of sea birds result in the deposition of carcasses, food scrapes, and guano on islands, which directly influences the availability of nutrition for terrestrial scavengers, as well as the productivity of primary producers on those islands (Stapp, Polis & Sânchez Pinero, 1999). To formalise an association between animal movement, conservation, and ecosystem function, Allen & Singh (2016) suggest a framework that incorporates the movement attributes of a species, the impact it has on the ecosystem, and the formulation of management plans. Given that animal movement is crucial for species survival and ecosystem function, climate change and land transformation are two of the major global changes that will impact animals in the Anthropocene (Sage, 2019).

Being forced to stay in inhospitable environments could lead to local population exterpation. In such scenarios, movement decisions such as dispersal and niche shifts become important and can lower extinction risk in the face of the climate change crisis (Román-Palacios & Wiens, 2020). However, adaptative response to climate change is not as simple as vulnerable animals shifting to suitable areas. Climate change affects the environmental cues that animals use for migration, increases their encounters with harsh climatic conditions that impact their physiology, and, in some cases, turn them sedentary (Seehacher & Post, 2015). Multiple examples of migratory birds advancing their arrival date at breeding sites in response to temperature shifts have already been documented (Kentie et al., 2018; Radchuk et al., 2019). Additionally, animals are known to respond to extreme events that climate change is likely to exacerbate, such as droughts (move to greener pastures), wildfires (move away from burnt areas), storms (move to refugia), and floods (move to highpoints) (Buchholz et al., 2019). The frequent occurrence and duration of extreme events could have implications for ecosystem functions that animals provide. For example, seed dispersal services provided by frugivores could be reduced as a consequence of global change, and this is expected to have consequences for plant species distribution (Mokany, Prasad & Westcott, 2014). Moreover, decrease of environmental predictability can impact crucial ecological processes that are dependent on animal movement, such as disease transmission, population dynamics of plants and animals, range distribution & interactions, and ecosystem functioning (Riotte-Lambert & Matthiopoulos, 2020).

Species moving in response to climate change can further be hindered by anthropogenic modifications that act as barriers (Chazdon et al., 2009). Increasing the size of urban areas can act as a barrier if suitable habitats are not available as stepping stones for animals to disperse (Leidner & Haddad, 2011). In addition to restricting dispersal, human activity can directly alter animal movement. Mammals living in areas with high human footprint reduced their median displacement by half compared to mammals in areas with a lower human footprint (Tucker et al., 2018). Animal populations that face rapid human-induced landscape changes, such as the development of roads or buildings, are predicted to be at risk of mortality since they have no *a priori* experience on how to respond to such modifications in the environment (Fahrig, 2007).

In the past decade, there has been a growing trend to review and synthesize studies in the field of movement ecology. For example, reviews tracking specific organisms such as bumblebees (Mola & Williams, 2019) or wild boar *Sus scrofa* (Morelle, Lehaire & Lejeune, 2014; Morelle et al., 2015) have highlighted technological advances and synthesized the state of knowledge for those species. Other reviews have identified gaps relating to our understanding of movement of a broad group of organisms, such as amphibians (Pittman, Osbourn & Semlitsch, 2014) and marine megafauna (Hays et al., 2016). There have also been reviews that examine the potential for animal tracking studies to contribute to conservation (Allen & Singh, 2016; Doherty & Driscoll, 2018; Katzner & Arlettaz, 2020). Additionally, there are reviews related to the increasingly advancing tracking technology (Sherub et al., 2017; Hofman et al., 2019), and the analytical methods to analyse fine-scale spatial data (Long & Nelson, 2013; Thums et al., 2018; Joo et al., 2020). Very few publications have explicitly reflected on the field at regional or country-wide scales, except for the Arctic region, where reviews have highlighted changes in terrestrial mammal migration patterns (Berteaux & Lai, 2021) and movements under threat of rapid climate change (Davidson et al., 2020).

Some of the most populated countries in the world, such as the Indian subcontinent are also biodiversity hotspots, and contain the largest populations of wild fauna, such as elephants, tigers, and other charismatic endangered wildlife. Species in countries such as India, face a double whammy of having to deal with global change in an environment with amongst the highest densities of people, and rapidly shrinking natural habitats. Given that movement ecology studies are essential to understand the ways in which animals respond to the environment, including anthropogenic modification and climate change, it is critical to synthesize the current state and advancements in the field for the country. Therefore, we take this opportunity to review how researchers have studied movement ecology in India. India is varied both in its geography and climate. The country hosts four biodiversity hotspots—from the Himalayas in the north and the Indo-Burma region in the northeast, to the Sundalands (Andaman & Nicobar Islands) in the southeast, and Western ghats in the southwest. The subcontinent varies climatically with glaciers in the north to deserts in the west, and rainforests in the northeast and southwest. India occupies 2.2% of the world’s land area but is home to 8.42% of all mammals, 13.66% of all birds, 8.05% of all reptiles, and 5.07% of all the amphibians (Ministry of Environment and Forests, Kalpavriksh, 2004). However, only 5.03% of the total land area of India falls under Protected Areas (PAs) (Forest Survey of India, 2019).

Two major environmental challenges that animals face in India are rapid and extensive changes to land cover and climate change. Although land-use changes have slowed since the 1980s with forest protection laws (Tian et al., 2014), certain regions, such as the Himalayas still face the threat of land-use change and fragmentation (Batar, Watanabe & Kumar, 2017). Changes in land use and land cover not only act as barrier for animal movement, but also contribute to changes in the climatic conditions of the region. Increasing urbanization in the southern parts of India increases surface temperature and seem to contribute to heavy rainfall events (Boyaj et al., 2020). Increasing agriculturalization in the western parts of India is likely to exacerbate desertification in the arid region (Varghese & Singh, 2016). Increasing urbanization has resulted in urban heat islands, in which urban areas are hotter than peripheral areas, in multiple Indian cities (Singh, Kikon & Verma, 2017; Swain et al., 2017; Puppala & Singh, 2021). Climate change projections from the country also indicate shifts in forest types (Ravindranath et al., 2006), and the forest vegetation itself being vulnerable in parts of western ghats, central India and upper Himalayas (Chaturvedi et al., 2011). Besides changes in vegetation that might require animals to disperse to suitable habitats, animals might also face the more immediate unpredictability of seasonality. The monsoon, a major seasonal phenomenon in the country, is expected to be delayed in the future by 10–15 days in many parts, and precipitation levels are expected to be 70% less than normal (Loo, Billa & Singh, 2015). These current and future environmental changes clearly show that it is crucial for animals to track changes and utilise movement strategies that facilitate their immediate survival, or even disperse into suitable habitats for sustained persistence.

This review aims to examine the last decade (2011–2021) of movement ecology studies in India and re-evaluates the extent of research gaps highlighted by Habib et al. (2014). By taking stock of all the animal tracking studies across India, we will also identify gaps in the taxa studied, landscapes where they are carried out, and research objectives. We conclude by identifying prospective research directions and proposing ways in which new studies could potentially contribute to the field of movement ecology, especially in an anthropogenically-changing India.

## Survey methodology

Movement ecology studies (peer-reviewed publications and reports) published between 2011–2021 were curated using two search engines: Google Scholar and Scopus. The primary keywords used for gathering these studies were ‘radio telemetry’, ‘satellite telemetry’, ‘GPS telemetry’, ‘home range’, ‘habitat use’, and ‘movement patterns’. Other keywords used in conjugation were ‘dispersal’ AND ‘distance’, ‘movement’ AND ‘animal’. All searches were screened with the keyword ‘India’ to narrow down the studies from the country. The criteria for selecting the publications from the search results were (1) the movement of the animal should be tracked within the country (2) individual animals (or group) must be identified with the tracking device attached to it, or through visual identification or genetic data (3) animals tagged outside the country but migrating into India were not included in the study, but global studies that include movement data from animals in India as well as other countries were included, and (4) movement of animals in laboratory conditions were not included. The search criteria for the Scopus database were restricted to ‘Title’, ‘Abstract’ and ‘Keywords’ of the publication. For the Google Scholar search engine, no such search restriction was imposed, but the search results were restricted to the first 10 pages (or 100 studies) (see Table S1 for details related to the search results). For additional information on the search process see the PRISMA 2020 flowchart and checklist in the supplementary (Page et al., 2021).

We summarised the published studies of animal movement from India between 2011–2021 and compared these with the previous review by Habib et al. (2014). Specifically, we discuss the number of publications during this period, species of animal taxa studied, region of the country the animals were tracked in, technology used for tracking and research goals of the publications.

## Results and discussion

### Number of publications in the field of movement ecology from India

**Figure 1:**
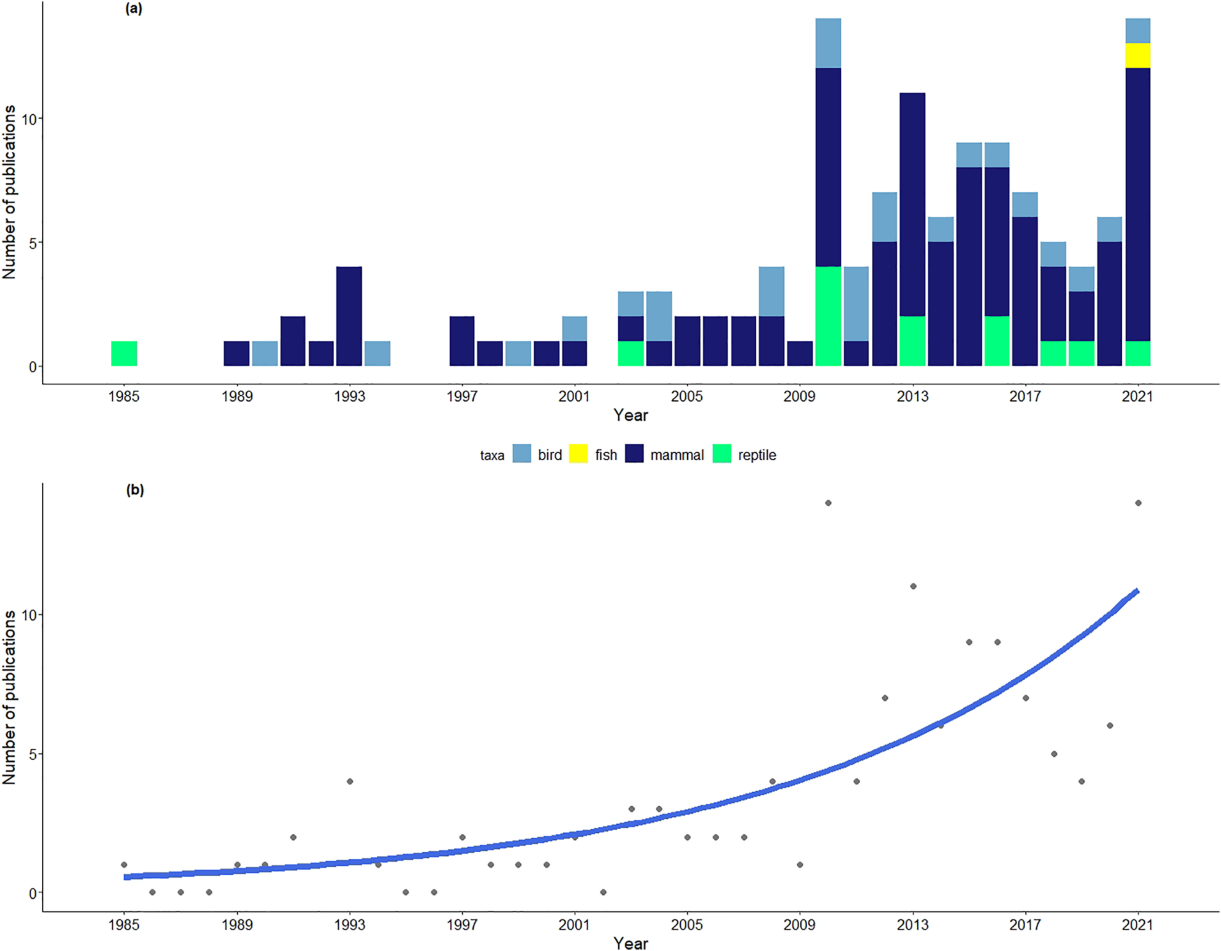
Number of publications in the field of movement ecology across years (1985–2021) from India. (A) Number of publications in the field of movement ecology across years (1985–2021) from India. The studies published between 1985–2010 were included in the review by Habib et al. (2014). (B) The trendline represents overall increase in number of publications in the field of movement ecology from India across years.

As expected, the number of publications (Figs. 1A and 1B) in the field of movement ecology from India has increased significantly over the years (Estimate = 0.082, *P* < 0.001, GLM: family=Poisson, link=log). This corresponds to the overall increase in the number of publications in the field due to advances in tools and technology—decreasing size and cost of tracking devices, the ability to collect finer-scaled spatio-temporal data and the development of appropriate and expansive statistical methods (Joo et al., 2018). The rapid increase in research publications in the field of movement ecology from India is also apparent when comparing the two different periods in which it is reviewed—Habib et al. (2014) reported 49 journal publications and reports between from 1985–2010 (26-year period) (Habib et al., 2014), and this study reports 82 between 2011–2021 (11-year period) (see Table S2).

### Taxa tracked across publications

**Figure 2:**
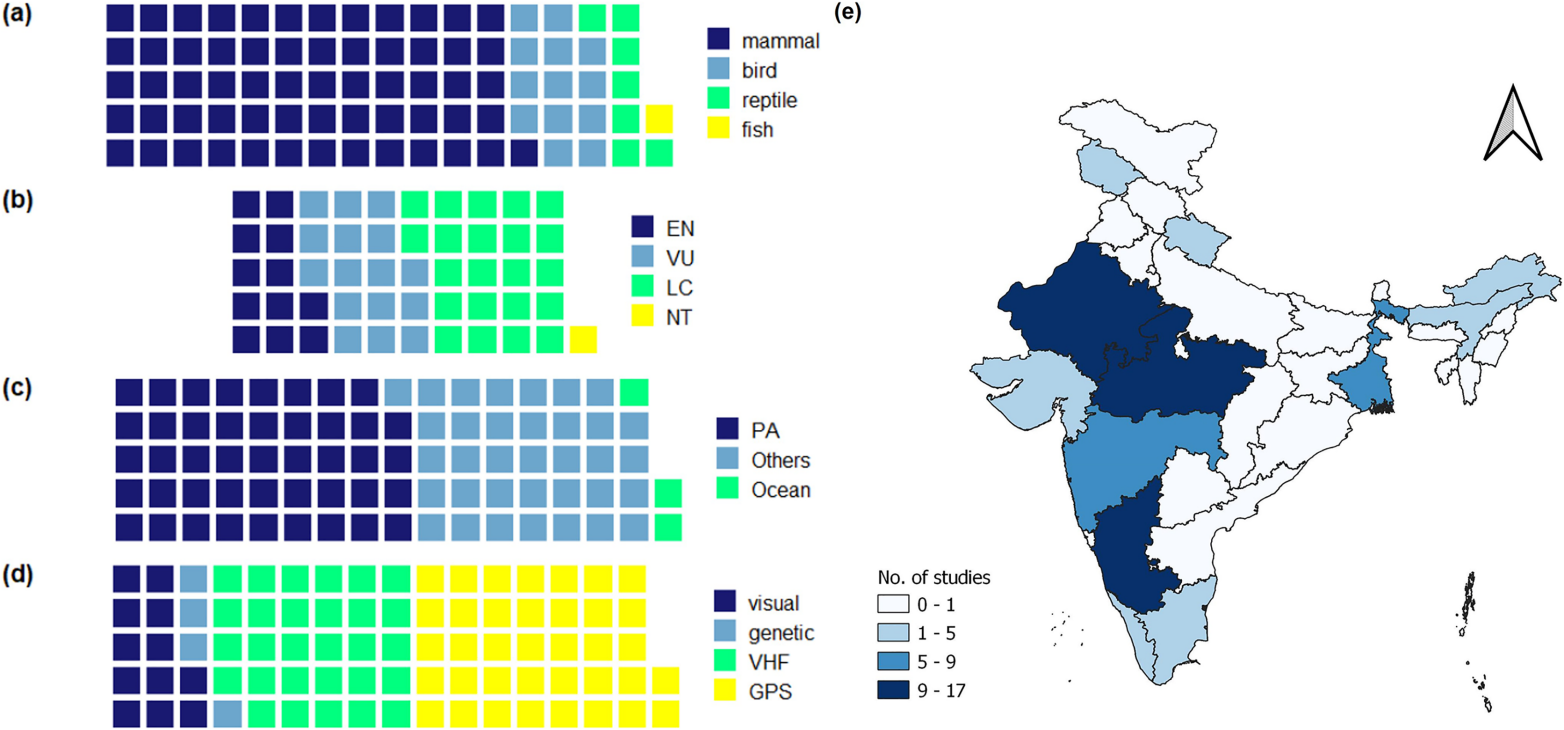
Different taxa tracked in movement ecology studies across India from 2011 to 2021, their IUCN status, methodology used for tracking, and geographic distribution of the studies. Between 2011–2021, from studies in India, (A) relative proportion of different taxa tracked in movement ecology studies (*n* = 82) (B) IUCN status of the 51 unique taxa tracked; EN, Endangered; VU, Vulnerable; NT, Near Threatened; LC, Least Concern (C) number of studies that tracked animal movement within terrestrial protected areas (PAs), in both protected areas and human-modified terrestrial landscapes (others), and in the ocean (D) methods employed for animal tracking, which include visual detection, non-invasive genetic tools, VHF and GPS collars. Shown also are the (E) geographical distribution of movement ecology studies in each state of India between 2011–2021 in India. In cases where a study is carried out in multiple states of the country the study is pseudo-replicated.

Amongst the research published in the past decade, mammals are the dominant taxa studied (76%; 62/82), followed by birds (13/82) and then reptiles (7/82) (Fig. 2A). Among mammals, the tiger, *Panthera tigris* alone accounted for 27% of the studies (22/82). Mammals were also the most dominant taxa tracked during the period from 1985–2010, and the tiger was the most dominant species among these as well. Due to this species bias, 78% of the animals tracked in the publications fall under the ‘Endangered’ or ‘Vulnerable’ threat categories of the International Union for Conservation of Nature (IUCN). However, among the 51 species tracked across all publications, 28 were in the ‘Endangered’ or ‘Vulnerable’ category while the rest were under ‘Least Concern’ or ‘Near Threatened’ (Fig. 2B). Only three of the 82 studies in this review tracked marine animals (Fig. 2C).

### Number of publications across the country

The review reveals that movement ecology studies are unequally distributed across the country (Fig. 2E). Most studies come from parts of Central and Western India—Madhya Pradesh (17) Rajasthan (13) and Maharashtra (eight). Majority of the studies on the two big cats, tigers, and leopards (86%) also come from these states. Other states that dominate the publications are Karnataka (10), West Bengal (seven), Tamil Nadu (five) and Assam (five). Very few published studies have tracked species in the north of India specifically in the Himalayan regions—a trend that was also identified by Habib et al. (2014).

### Methods for recording movement

The technology used for tracking animals in the studies included in this review fell into a few broad categories. Majority of studies (80%; 66/82) used Very High Frequency (VHF) radio transmitters, or GPS transmitters attached to animals for tracking movement (Fig. 2D). GPS transmitters (used in 37/82 studies) included devices that transmit spatial data to hand-held receivers, mobile networks, or satellites.

Non-invasive methods such as identifying individuals through visual encounters, through photographs from camera traps, or through genetic data from fur or fecal samples formed the rest of the movement studies (16/82). Non-invasive methods use unique intrinsic markers of individuals, such as coat patterns and genetic information for infer movement and displacement across the landscape (Hobson & Ryan Norris, 2008). For example, images from camera traps were used to identify and estimate the home ranges of leopards (Kumbhojkar et al., 2020), dispersal distance of tigers (Singh et al., 2013) and space acquisition of vacated sites in tigers (Singh et al., 2020). Genetic evidence from tigers have been crucial to infer movement (Reddy et al., 2012) and dispersal patterns (Gour et al., 2013), and to determine whether tiger populations were connected across human-modified landscapes in central India (Joshi et al., 2013). Studies on the home range of primates have used visual identification to record movement of the groups. For example, the home range of Eastern Hoolock Gibbon *Hoolock leuconedys* (Sarma & Kumar, 2016), Lion-tailed macaques *Macaca Silenus* (Erinjery, Kavana & Singh, 2015; Santhosh et al., 2015) and Rhesus macaque *Macaca mulatta* (Sengupta, McConkey & Radhakrishna, 2015) were recorded by observers using hand-held GPS devices.

### Research goals across publications

#### Home range and monitoring

Over the last decade, the research goal in 60% (49/82) of the publications were predominantly centred on measuring the home ranges and/or habitat use of target species (see Table S2). Such a dominance towards examining species home range in studies was also observed by Habib et al. (2014). Unlike in the studies before 2010, recent studies investigated home-range sizes for different purposes (see Table S2). Several studies were designed to compare home range sizes across species based on body size to test allometric-based hypotheses (Namgail et al., 2014; Noonan et al., 2020; Katna et al., 2021), or for the same species across seasons (Erinjery, Kavana & Singh, 2015; Jha et al., 2018; Katna et al., 2021), or between sexes (Rana, Kalsi & Burra, 2012; Naha et al., 2016; Singh et al., 2016). These studies provide new insights, such as the observation that male tigers living in the mangroves of Sundarban had an average home range nearly twice that of females (Naha et al., 2016). Or that the home range size of the Bengal Florican *Houbaropsis bengalensis* tagged in Uttar Pradesh, India and Nepal, is greater in the non-breeding season than in the breeding season (Jha et al., 2018). Notably, newer studies have used home range data to understand how animals utilise human-modified landscapes (Kumbhojkar et al., 2020; Katna et al., 2021; Naha et al., 2021) or respond to new areas after translocation (see section on *Movement ecology for conservation management* below).

Besides information on the ranging behaviour of animals and how they use the landscape, recent studies have provided other valuable insights on target species through continuous monitoring of movement. For example, fecal glucocorticoid metabolites collected from tracked tigers show elevated stress levels in response to anthropogenic disturbance (Bhattacharjee et al., 2015). Continuous animal tracking also reveals information on inter-birth intervals in female tigers (Sadhu et al., 2017) which is essential for understanding the population dynamics of this endangered species.

#### Animals tracked in human-modified landscapes (HMLs)

Most animals are not confined to protected areas (PAs) and are likely to move in and out of HMLs. Among the publications since 2011, 54% (44/82) of studies were from animals tracked in PAs, while 43% (35/82) were of either animals that shuttled between PAs and HMLs or were moving exclusively in HMLs (Fig. 2C). In HMLs, animals make decisions on whether to use the remnant native vegetation or the human-modified agricultural areas. For example, in a semi-arid landscape of western India, the Indian fox *Vulpes bengalensis* range mainly in fragmented native grasslands, whereas Golden jackals *Canis aureus* and Jungle cats *Felis chaus* readily utilise plantations and other human-modified areas of the landscape (Katna et al., 2021). For the Lesser false vampire bat *Megaderma spasma*, remnant forest patches in the western Ghats were selected over plantations (Prakash et al., 2021). But such habitat selection might change across seasons. The Leopard *Panthera pardus* in a fragmented landscape in eastern India selects dense forest patches in the wet season but was found using plantations during the dry season (Naha et al., 2021). Besides habitat use, movement patterns and decisions of animals also differ based on whether the animal is found within the PAs or outside. For example, the hourly displacement of tigers outside PAs is greater than within PAs (Habib et al., 2021). Another challenging movement that large carnivores carry out in their lifetime is long-distance dispersal to suitable habitats, which in India, involves travelling through human-modified landscapes. Individual tigers can disperse nearly ~700 km (established through genetic analysis) but their dispersal is negatively affected by human settlements and roads (Joshi et al., 2013).

#### Consequences of animal movement

Another major development in the field of movement ecology has been to relate animal movement with consequences for the biotic community. Such studies are especially relevant for frugivores and the seeds of plant species they disperse. Naniwadekar et al. (2019) showed that the average seed dispersal distance (computed from animals’ movement & gut passage time) of the Great hornbill *Buceros bicornis* was lower than the dispersal distance of the Wreathed hornbill *Rhyticeros undulatus*. These birds also differ in distances they travel in the breeding and non-breeding season, which can directly influence dispersal distance (Naniwadekar et al., 2019, 2021). Studies like these have consequences for the management of invasive plant species in an area, since seed dispersal patterns are affected by the distance and movement strategies of dispersers, such as birds that consume the fruits (Ramaswami et al., 2016). Similarly, data from GPS-telemetry on Asian elephants in tropical moist forests of northern West Bengal were used to model fruit dispersal; this study showed that elephants were more effective dispersers of three tropical large-fruited species compared to other large mammals, such as bovids (Sekar, Lee & Sukumar, 2015, 2017). The seed dispersal function of Asian elephants may be essential for the persistence of these tree species when impacted by habitat fragmentation and climate change (Sekar, Lee & Sukumar, 2015, 2017). Humans can also affect seed dispersal patterns. Rhesus macaque *Macaca mulatta*, had shorter ranges when provisioned by humans than when they were not provisioned, resulting in shorter dispersal distances for the plant seeds the animals consumed (Sengupta, McConkey & Radhakrishna, 2015).

#### Movement ecology for conservation management

Studies examining the home range of animals, or habitat use in natural areas provide key information about what a species requires for its survival. Such information provides baseline data for conservation plans in a protected landscape. Particularly relevant among these are studies that track the movement of a reintroduced or translocated animal to understand how they use new environments (Jha, 2011; Sankar et al., 2013; Sarkar et al., 2016; Bhardwaj et al., 2021). Home range information has been used to monitor reintroduced or translocated species—tigers (Bhardwaj et al., 2021), leopard (Mondal et al., 2013) one-horned rhino *Rhinoceros unicornis* (Barman et al., 2014; Dutta et al., 2017), sloth bear *Melursus ursinus* (Arun et al., 2021) and translocated king cobra *Ophiophagus hannah* (Barve et al., 2013) to understand how they were acclimatising to their new environments. The recent introduction of the cheetah *Acinonyx jubatus jubatus* to Kuno National Park in India will also utilise movement data from satellite collars to determine home-range and habitat selection of these individuals in their new environment (see Table S3). When tigers were re-introduced to Panna Tiger Reserve, movement data across generations were key to understanding how tigers were using human-dominated areas and what factors could result in human-wildlife conflict (Kolipaka et al., 2018). Using animal tracking to understand and mitigate human-wildlife conflict is especially relevant in the case of elephant-human conflict, in which continuous monitoring and rapid response helps reducing crop loss or, threats to humans (Venkataraman et al., 2005) and prevents elephant mortality when they cross roads or railways (Datta et al., 2016). Asian elephants are being monitored in many parts of the country by the state forest departments to reduce conflict—from the state of Karnataka in the south to Uttarakhand in the north, and Odisha, West Bengal, Tripura and Assam in the east (see Table S3 for sources to newspaper articles), and the scientific findings of these are forthcoming.

Another significance of studying movement is the conservation implication it has for marine animals. Recent studies from India have tracked the post nesting migration routes of Olive ridley turtles *Lepidochelys olivacea* in Odisha coast (Behera et al., 2018) and Leatherback turtles *Dermochelys coriacea* from Andaman Islands (Swaminathan, Namboothri & Shanker, 2019). Such studies are not just useful for identifying the external factors such as sea surface temperature and chlorophyll concentration that influence turtle movement (Behera et al., 2018) but are also valuable to identify regions along the migratory route where protective measures, such as programs to reduce fishing pressure, can be implemented (Swaminathan, Namboothri & Shanker, 2019).

Besides reducing conflict, recent efforts to track animals in human-modified landscapes (Habib et al., 2021; Katna et al., 2021; Naha et al., 2021) are crucial to identify corridors and manage connectivity that facilitate species dispersal across landscape matrices. Facilitating connectivity, based on actual movement data, is an essential step towards enabling successful dispersal considering potential alterations to environments.

#### Animal movement in response to climate change

No study from India has explicitly explored how climate change might affect animal movement behaviour. Recent studies that have examined seasonal shifts in animal movement (Namgail et al., 2011; Erinjery, Kavana & Singh, 2015) provide critical indications of how temperature and precipitation patterns influence movement strategies. Newer studies are starting to also examine how species distributions are likely to shift with future climate change predictions (Jose & Nameer, 2020; Raman et al., 2020). For example, the Indian Peafowl *Pavo cristatus* is predicted to expand its distribution in response to changes in temperature and precipitation (Jose & Nameer, 2020). This species-level prediction does not, however, reveal how individuals may respond to climate change within their lifetime.

One particularly significant movement decision related to global change is migration patterns. Since animals use environmental cues to time migration and navigation (Seehacher & Post, 2015), migrating animals are expected to be amongst the first responders to changing climatic conditions. Recently published studies from India have identified migratory routes of Black kite *Milvus migrans*, (Kumar et al., 2020), Lesser florican *Sypheotides indica* (Sivakumar et al., 2016), Bar-headed geese *Anser indicus* (Hawkes et al., 2011; Mohit et al., 2011) and Leatherback sea turtle *Dermochelys coriacea* (Swaminathan, Namboothri & Shanker, 2019). Most studies used satellite transmitters to track animal migrations, however there are other techniques, such as quantifying stable isotopes from animal tissue to infer movement patterns (Hobson & Ryan Norris, 2008). Stable isotopes are used for tracking migration and space use patterns in a range of animals, including marine species (Ramos & González-Solís, 2012) and terrestrial species (Rubenstein & Hobson, 2004). However, we found no publications from India thus far that uses stable isotopes to answer questions relating to movement of animals. More such studies on migratory routes combined with long-term monitoring of arrival and departure times will provide the necessary information about climate-induced changes to movement decisions of animals, such as birds or butterflies. It would also be relevant to study migration patterns across years in relatively long-lived species, such as mammals (*e.g*., elephants) or marine taxa (*e.g*., sea turtles), to determine how individuals cope with the changes in the environment during their lifetime.

## Conclusion

### Potential research directions for India in the field of movement ecology

Given the rapid growth and current state of the field of movement ecology in India, the possibility for generating new knowledge is abundant. This literature review, spanning movement ecology research in India over the last 11 years, shows the disproportionate focus on some taxa (*e.g*., tigers) and some metrics of movement (*e.g*., home range). We take this opportunity to call for a broadening of the scope of movement ecology studies, bringing forth the most urgent needs of the field. We also seek to identify where the unique biogeography and biodiversity of India can provide interesting opportunities for research in this field (Table 1).

**Table 1:** Prospective research directions in the field of movement ecology and potential questions that can be investigated in India.

No.	Research topics	Potential questions to address
1.	Tracking species across seasons and years 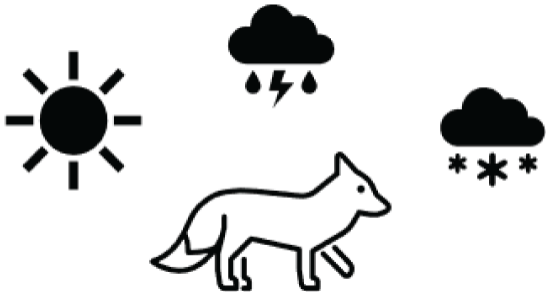	How do animals in different biogeographic zones track climatic changes?
2.	Tracking species across different landscapes 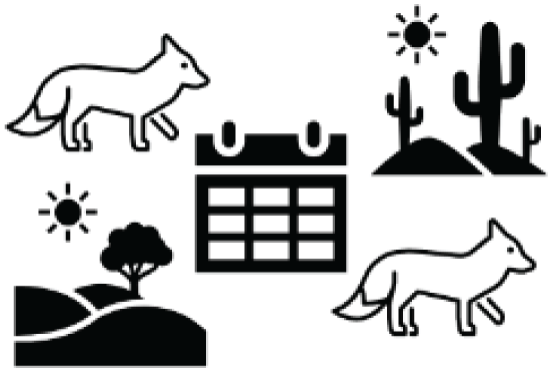	How generalised are species responses, or are movement strategies driven mainly by the immediate environment?
3.	Tracking many individuals 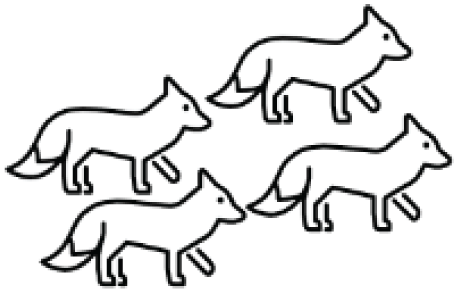	How do conspecific interactions influence individual movement decisions?
4.	Tracking species outside protected areas 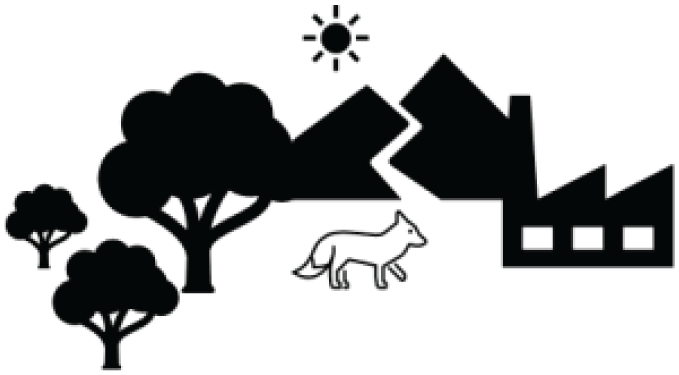	What are the strategies and limitations that enable animals to survive with anthropogenic alterations to their environment?
5.	Tracking diverse taxa in the same area 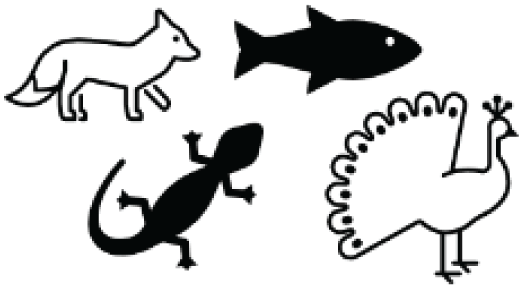	How do species traits individually or in combination affect the movement decisions of a taxa?
6.	Tracking smaller taxa 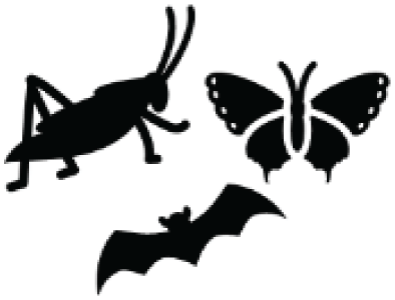	How do smaller animals make movement decisions, from daily foraging across habitats to long-distance migration across continents?
7.	Tracking marine and freshwater taxa 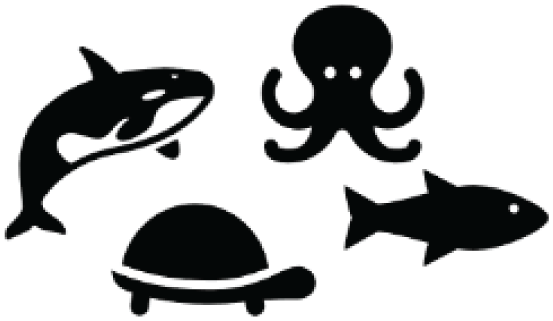	What are the migratory patterns of marine taxa? How do humans impact their movement? Are there eco-sensitive areas that need additional protection?
8.	Tracking animals to reduce human-wildlife conflict 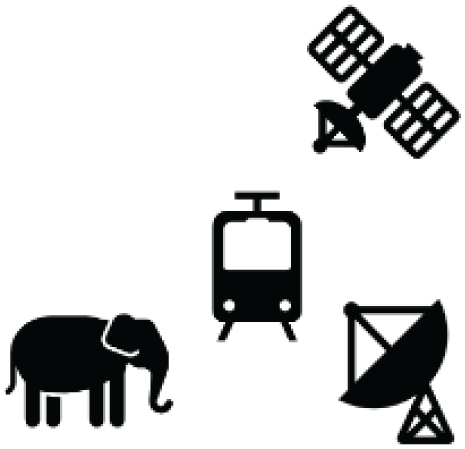	Are there patterns of animal movement that enable proactive and anticipatory action to mitigate human-wildlife conflict?
9.	Tracking animals and ecological processes 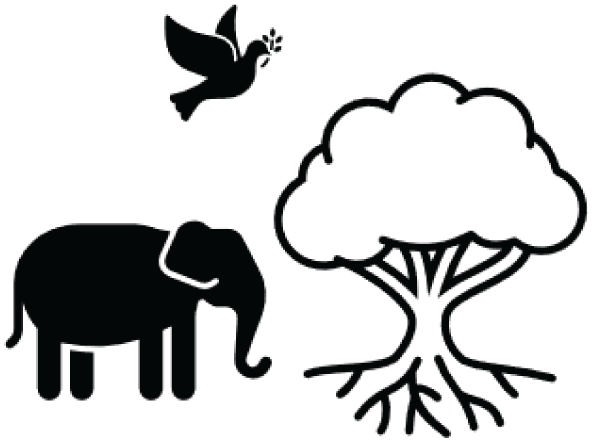	How does animal movement impact various ecological processes, such as seed dispersal or pollination? How does global change affect these crucial functions?
10.	Tracking animals with different sensors 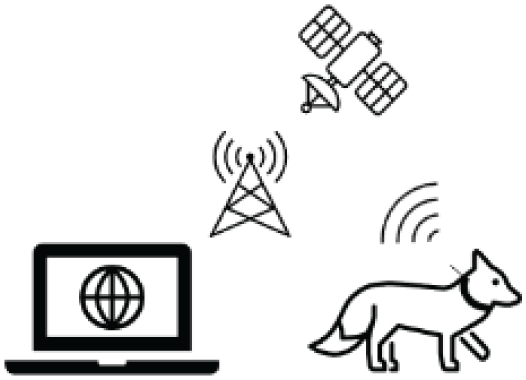	What environmental variables best track changing conditions and how do they influence animal movement?

1. There is considerable insight that can be gained from tracking species over long periods of time, such as multiple seasons and years. Movement decisions of animals are driven in large part by distribution and availability of resources, which change with seasonality (Abrahms et al., 2021). Seasonality in India is influenced by various spatially explicit factors. Parts of India receive the bulk of the monsoon rains and these regions can be divided into the wet and dry seasons. Southern parts of the country are tropical, while the northern parts are sub-tropical to temperate. Some parts of India are also projected to face greater effects of climate change than others, compounding the impacts on environments. Understanding whether and in what ways animals can respond to natural and human-induced climatic shifts across years, through long-term studies, will enable us to determine how quickly animals, resources and their drivers can respond.
2. Patterns of resource distribution varies across landscapes, and thus the same species in different environments are expected to differ in their movement decisions (Shepard et al., 2013). Monitoring animals across different landscapes enables us to infer the significant drivers of movement and their variation, to determine how much of movement strategies are inherent to the species, and how much are driven by local environmental conditions. From evergreen forests in the southwest and northeast to arid deserts in the west and snow-clad mountains in the north, India possesses diverse landscapes. Each landscape consists of species adapted to the region, but they also share some species. Thus, tracking the same species in different environments across seasons, is a powerful approach, especially in the context of global change.
3. The movement strategies of individuals are not only driven by environmental conditions but are also influenced by interactions with conspecifics. Simultaneous tracking of many interacting individuals in an area will provide insights into how social interactions influence decision-making and space-use patterns (reviewed in Westley et al., 2018). Until now, most animal tracking in India has been confined to understanding the extent of space-use patterns of a few individuals of a species. Multiple individuals are rarely tracked together, and thus, the contribution of group members or conspecifics to movement decisions is an exciting research direction.
4. Natural spaces for animals are reducing (Hirt et al., 2021) and animals are increasingly forced to utilise landscapes modified by humans. Many species might curtail their movement altogether in these altered landscapes (Tucker et al., 2018). Such impedance to movement may affect the animal’s dispersal (Chazdon et al., 2009) and in the long term, species distribution. India has a population of ~1.35 billion people spread across urban and rural areas, and thus, human-animal encounter rates are high. Dedicated wildlife habitats in India are fragmented and are flanked by either residential, agricultural, and/or industrial developments. Tracking species outside protected areas will generate a stronger understanding of the strategies and constraints that enable animals to survive in an era of global change, which includes a moving animal being able to tackle both climate change and land transformation (Sage, 2019).
5. The movement behaviours and strategies of animals are influenced by multiple intrinsic factors, such as size (Jetz et al., 2004), morphology (Börger et al., 2020), and cognitive ability (Kashetsky, Avgar & Dukas, 2021). To make generalizable predictions of animal movement that examine the effects of intrinsic differences while controlling for extrinsic or environmental factors (Nathan et al., 2008), tracking different kinds of taxa in the same area is a powerful approach. India hosts ~400 mammals, ~1,400 birds, and ~600 reptiles and amphibian, and thus the opportunities to track diverse taxa that play key functional roles within an ecosystem is immense.
6. Obtaining movement data from smaller vertebrates and invertebrates is a challenge, given that bio-loggers must be miniature and weigh a fraction of the animal’s weight. Animals as small as bumblebees (200–450 mg; Hagen, Wikelski & Kissling, 2011) and butterflies (300–700 mg; Fisher, Adelman & Bradbury, 2020), have been tracked to determine flight paths, space use, and migration patterns. Other technological advances with projects such as ICARUS (Belyaev et al., 2020; Jetz et al., 2022) and wireless network sensors (Ripperger et al., 2020) have made the fine-scale tracking of numerous small taxa more feasible today. Given that the technology to track the movement of smaller animals is rapidly advancing, future studies that track the movement of insects, smaller mammals, and birds in India, will provide key insights on species that have not been tracked before.
7. The marine ecosystem offers an opportunity to address many key ecological questions—from the influence of memory or learning, and social interactions, to prey distribution, and the impact of global change (Hays et al., 2016). Marine animals with sensors can potentially act as sentinels and record environmental variables in regions in the ocean not commonly sampled, which will allow us to better understand climate and ocean variability (McMahon et al., 2021). With a coastline of ~7,500 km, there is immense potential to track the movement of various marine animals to provide relevant information for the conservation of species and ecologically sensitive marine zones of India.
8. Animal tracking has a direct application in regions with human-wildlife conflict. Tracking animals is not only useful for understanding animal movement, but it helps reduce conflict by sending early warning signals to alert people to the location of the animal so they can take pre-emptive action (Venkataraman et al., 2005). In India, animal tracking studies on large carnivores (such as tigers and leopards), or herbivores (such as elephants, wild pigs *Sus scrofa*, and nilgai *Boselaphus tragocamelus*) can help mitigate direct and indirect human-wildlife conflict. Animal tracking information in combination with early warning signal alerts, can potentially prevent human deaths and infrastructure damages (Kumar & Raghunathan, 2014), reduce agricultural crop damages, and avoid railway collisions of wildlife (Datta et al., 2016). Such studies can also determine barriers and corridors to movement (Joshi et al., 2013). Additionally, citizen-science information of animal locations would be a valuable complement to telemetry data, enabling a better understanding of animal movement decisions, especially of species prone to human-wildlife conflict.
9. Animal tracking studies are crucial for understanding how movement affects ecosystem processes in a landscape. Movement of frugivorous birds (Ramaswami et al., 2016; Naniwadekar et al., 2019, 2021) and mammals (Sekar, Lee & Sukumar, 2015) have implications for where plants species disperse their seeds and their future survival. Loss of this dispersal service will directly affect the capacity of plant species to respond to changing climatic conditions (Mokany, Prasad & Westcott, 2014). Landscape fragmentation also affects the movement of bees and butterflies, and therefore impacts plant pollination (Hadley & Betts, 2012). Additionally, megaherbivores like an elephant trampling through a forest can engineer ecosystems by changing vegetation patterns (Haynes, 2012). Given the latest advances in animal tracking devices (see (6) and (10) in this section) it is now possible to study how animal movement influences ecological processes in different climatic regions across India.
10. Technological advances have spearheaded the field of movement ecology over the last two decades in ways that enable the tracking of species as well as their environments (Kays et al., 2015; Sherub et al., 2017). Innovations in technology, methods to analyse big data (Thums et al., 2018) and sensors that record different kinds of information (Sherub et al., 2017), have all expanded the scope to ask newer ecological questions related to the movement decisions of animals. This also allows for the standardisation of bio-logging devices for conservation management use (Sequeira et al., 2021). Additionally, collared animals themselves can now be used to collect fine-scaled data on the environments they utilise (Börger et al., 2020; Thaker et al., 2019). With the available tracking technologies, it’s now possible to understand and mitigate the impacts of global change, a direction that India is primed to pursue. Going beyond the focus of individual species or specific geographic regions in India, we can now track animal movement with environmental sensors that also track temperature, wind pressure, sound, and several other key parameters.

### Limitations to this review and to the growth of movement ecology in India

To the best of our knowledge, we have conducted an exhaustive search of animal movement studies carried out in India over the last 11 years. We acknowledge that some studies may have been missed, especially if these were not published in peer-reviewed journals listed on SCOPUS or Google Scholar. For example, many state forest departments collar and track species of interest for general monitoring and to mitigate wildlife conflict. These data are typically not available for public access or published in scientific journals. Additionally, studies with low sample sizes are more likely summarised in reports to granting agencies or forest departments, which are also not accessible. Therefore, the taxa that we identify as having been collared or tagged over the last decade is likely to be missing species. There has, however, been a new push to tagging and tracking animals and many of these initiatives have been announced in newspapers or social media (see Table S3), and so the field of movement ecology in India is set to see a rapid period of growth. Overall, our review effectively captures the general trends and approaches in the field of movement ecology in the country and has allowed us to identify research gaps and future research directions. Some of the potential research directions that we propose, for example monitoring animal movement in human modified landscapes to reduce wildlife conflict, have already been initiated. Similarly, the tagging of migratory species, such as the endangered Great Indian Bustard, is also ongoing and is of urgent national importance. Although not included here, these studies reflect new and necessary directions for this field.

Among the shortcomings identified in the previous review from India, by Habib et al. (2014), a lack of animal tracking studies from the northern part of India, an emphasis on certain research goals (*e.g*., home-ranges and habitat selection), and the need for a centralised system to ease the permission to capture and collar animals, were highlighted. While there appears to be a positive trend to address some of these issues, most of the deficiencies still exist. One way to address these gaps is by forming a biologging group/forum/conclave that brings together both experts and stakeholders from various research institutions, state forest departments, and conservation organizations to share information, technology, and collaborate on future projects across the county. Collaborations between research institutions and government agencies might help reduce the difficulties in obtaining research permits for capturing and collaring animals and may directly facilitate conservation management goals. Additionally, we urge researchers from India to adopt an open data policy whenever possible, which would involve uploading animal movement data, both published and ongoing, to global databases, such as Movebank (Kays et al., 2022). By joining the global initiative of movement ecology, researchers from India have larger opportunities to collaborate on global-level analyses of animal movement. In the rapidly changing Anthropocene, where anthropogenic disturbance and climatic change are irreversibly altering the Earth, collaborative global approaches to tag, track, and understand animal movements is essential to understand species responses and to mitigate impacts on crucial ecosystem functions.

## Supplemental Information

10.7717/peerj.14401/supp-1 Supplemental Information 1 Search results from keywords, list of publications, and ongoing studies in the field of movement ecology.

10.7717/peerj.14401/supp-2 Supplemental Information 2 Prisma Checklist for the systematic review.

10.7717/peerj.14401/supp-3 Supplemental Information 3 Prisma flow diagram of the survey and the final number of studies retrieved for the review.

## Additional Information and Declarations

### Data Availability

The following information was supplied regarding data availability: The data is available in the Supplemental File.
