# Supplementary material for: Animal movement ecology in India: insights from 2011–2021 and prospective for the future
Source: PeerJ. 2022 Dec 13;10:e14401. doi: 10.7717/peerj.14401 (PMC9756863; doi:10.7717/peerj.14401)
Supplement: Supplemental Information 1 [file peerj-10-14401-s001.docx]

# Supplementary Information

# Manuscript Title

Animal movement ecology in India: insights from the last decade (2011-2021) and prospective for the future.

**Authors**

Harish Prakash^1, #^, R. Suresh Kumar^2^, Bibhuti Prasad Lahkar^3^, Raman Sukumar^1^, Abi Tamim Vanak^4,5,^ , Maria Thaker^1,#^

**Affiliations**

1. Centre for Ecological Sciences, Indian Institute of Science, Bengaluru, India

2. Department of Endangered Species Management, Wildlife Institute of India, Dehradun, India

3. Aaranyak, Guwahati, India

4. Ashoka Trust for Research in Ecology and the Environment, Bengaluru, India

5. School of Life Sciences, University of KwaZulu-Natal, Durban, South Africa

# - except for the first and senior author, all the other authors are listed alphabetically

**Corresponding Author:**

Harish Prakash, Maria Thaker

Centre for Ecological Sciences, Indian Institute of Science, Bengaluru-560 012, India

Email: [harishp@iisc.ac.in](mailto:harishp@iisc.ac.in); [harishprakashhp@gmail.com](mailto:harishprakashhp@gmail.com), [mthaker@iisc.ac.in](mailto:mthaker@iisc.ac.in)

This document contains three tables and references associated with Table 2.

***Table S1.*** *Number of search results from each keyword used in database, Scopus and search engine, Google Scholar* as of July 2022****. *****Total hits for Google Scholar search engine were restricted to first 100 publications.*

| ***No.*** | ***Keywords used*** | ***Scopus*** | | | ***Google Scholar**** | |
| --- | --- | --- | --- | --- | --- | --- |
|  |  | ***Total hits*** | ***No. of selected publications*** | ***Percentage*** | ***No. of selected publications*** | ***Percentage*** |
| 1. | ‘radio telemetry’, ‘India’ | 13 | 10 | 77% | 17 | 17% |
| 2. | ‘GPS telemetry’, ‘India’ | 4 | 2 | 50% | 13 | 13% |
| 3. | ‘satellite telemetry’, ‘India’ | 13 | 5 | 38.5% | 10 | 10% |
| 4. | ‘home range’, ‘India’ | 85 | 11 | 13% | 21 | 21% |
| 5. | ‘habitat use’, ‘India’ | 126 | 12 | 9.5% | 12 | 12% |
| 6. | ‘movement patterns’, ‘India’ | 57 | 9 | 15.8% | 13 | 13% |
| 7. | ‘dispersal’, ‘distance’, ‘India’ | 142 | 10 | 7% | 10 | 10% |
| 8. | ‘movement’, ‘animal’, ‘India’ | 270 | 12 | 4.4% | 9 | 9% |

**Possible search result biases**

We acknowledge that our keyword search list (listed in Table 1) and criteria for selection (see survey methodology) have limitations and might not cover the entire gamut of studies published in the field of movement ecology from India between 2011-2021. For example, studies that examine habitat use of species were restricted to ones that identified and tracked individuals (or groups) repeatedly as compared to studies that recorded the presence/absence of the species in a habitat but did not identify the individual. Additionally, there might be shortcomings in the information we extracted from the selected publications. The objectives, for example, that we gathered from the publication (see supplementary Table 2) are the main finding of the study, but not an exhaustive list of all the movement metrics the publication reported. Finally, the trends and the future research directions we recommend are based on the literature we curated as well as the accessibility of publications in the databases. It is not possible for us to assess if unpublished and ongoing studies are similar or different from the field trends we report.

***Table S2****. Taxa tracked and the objectives of* ***82*** *published studies from India in the field of movement ecology between 2011-2021.*

| **No.** | **Common Name** | **Species Name** | **Title** | **Study objective** | **Source** |
| --- | --- | --- | --- | --- | --- |
| 1 | Ruddy Shelduck | *Tadorna ferruginea* | Ruddy Shelduck *Tadorna ferruginea* home range and habitat use during the non-breeding season in Assam, India | Home range, Habitat use | (Namgail et al., 2011) |
| 2 | Bar-headed geese | *Anser indicus* | The trans-Himalayan flights of bar-headed geese (*Anser indicus*) | Migration | (Hawkes et al., 2011) |
| 3 | Bar-headed geese | *Anser indicus* | Satellite tracking of Bar-headed Geese *Anser indicus* wintering in Uttar Pradesh, India | Migration | (Mohit et al., 2011)Mohit et al., 2011 |
| 4 | Grey Fracolin | *Francolinus pondicerianus* | Home range comparison of male and female Grey francolin (*Francolinus pondicerianus*) using radiotelemetry | Home range | (Rana, Kalsi & Burra, 2012) |
| 5 | Pallid Harrier | *Circus macrourus* | Broad wintering range and intercontinental migratory divide within core population of the near-threatened pallid harrier | Migration, range | (Terraube et al., 2012) |
| 6 | Garganey | *Spatula querquedula* | Space use of wintering waterbirds in India: Influence of trophic ecology on home-range size | Home range of migratory birds | (Namgail et al., 2014) |
|  | Northern shoveler | *Spatula clypeata* |  |  |  |
|  | Eurasian wigeon | *Mareca penelope* |  |  |  |
|  | Gadwall | *Mareca strepera* |  |  |  |
|  | Northern pintail | *Anas acuta* |  |  |  |
| 7 | Bar-headed geese | *Anser indicus* | Tracking the Movement Pattern of Bar-Headed Goose *Anser indicus* Captured from the Gharana Conservation Reserve, India | Home range | (Mahar et al., 2015) |
| 8 | Red-vented bulbul | *Pycnonotus cafer* | Dispersal by generalist frugivores affects management of an invasive plant | Seed dispersal distance | (Ramaswami et al., 2016) |
|  | Red-whiskered bulbul | *Pycnonotus jocosus* |  |  |  |
| 9 | Lesser florican | *Sypheotides indica* | A Study on Ecology and Migration of the Lesser Florican (*Sypheotides indica*) in Western India Using Satellite Tracking Techniques | Home range, migration, habitat use | (Kuppusamy et al., 2017) |
| 10 | Bengal Florican | *Houbaropsis bengalensis* | Distribution, movements, and survival of the critically endangered Bengal Florican *Houbaropsis bengalensis* in India and Nepal | Home range, survival | (Jha et al., 2018) |
| 11 | Great Hornbill | *Buceros bicornis* | How far do Asian forest hornbills disperse seeds? | Seed dispersal distance | (Naniwadekar et al., 2019) |
|  | Wreathed Hornbill | *Rhyticeros undulatus* |  |  |  |
| 12 | Black-eared Kite | *Milvus migrans* | GPS‑telemetry unveils the regular high‑elevation crossing of the Himalayas by a migratory raptor: implications for definition of a “central Asian flyway” | Migration | (Kumar et al., 2020) |
| 13 | Great Hornbill | *Buceros bicornis* | Roost site use by Great (*Buceros bicornis*) and Wreathed (*Rhyticeros undulatus*) Hornbill and its implications for seed dispersal | Seed dispersal distance, Roost site use, home range | (Naniwadekar et al., 2021) |
|  | Wreathed Hornbill | *Rhyticeros undulatus* |  |  |  |
| 14 | King Cobra | *Ophiophagus hannah* | Habitat use of King Cobra (*Ophiophagus hannah*) in a heterogeneous landscape matrix in the tropical forests of the Western Ghats, India | Habitat use | (Rao et al., 2013) |
| 15 | King Cobra | *Ophiophagus hannah* | A preliminary study on translocation of “rescued” King Cobras (*Ophiophagus hannah*) | Home-range, distance post translocation | (Barve et al., 2013) |
| 16 | Travancore Tortoises | *Indotestudo travancorica* | Fine Scale Habitat Selection in Travancore Tortoises (*Indotestudo travancorica*) in the Anamalai Hills, Western Ghats | Habitat selection | (Deepak, Noon & Vasudevan, 2016) |
| 17 | Tricarinate Hill-turtle | *Melanochelys tricarinata* | Modelling fine scale movement corridors for the Tricarinate Hill turtle | Fine scale movement, habitat connectivity | (Mondal et al., 2016) |
| 18 | Olive Ridley Turtles | *Lepidochelys olivacea* | Movements of Olive Ridley Turtles (*Lepidochelys olivacea*) in the Bay of Bengal, India, Determined via Satellite Telemetry | Migration | (Behera et al., 2018) |
| 19 | Leatherback Turtles | *Dermochelys coriacea* | Tracking Leatherback Turtles from Little Andaman | Migration | (Swaminathan, Namboothri & Shanker, 2019) |
| 20 | Russell's viper | *Daboia russelii* | Proximity between humans and a highly medically significant snake, Russell’s viper, in a tropical rural community | Proximity to humans | (Glaudas, 2021) |
| 21 | Whale Sharks | *Rhincodon typus* | First Insights Into the Horizontal Movements of Whale Sharks (*Rhincodon typus*) in the Northern Arabian Sea | Horizontal movement | (Arrowsmith et al., 2021) |
| 22 | Red Panda | *Ailurus fulgens* | Chapter 25 - Release and Reintroduction of Captive-bred Red Pandas into Singalila National Park, Darjeeling, India | Monitoring reintroduction | (Jha, 2011) |
| 23 | Tiger | *Panthera tigris* | Home ranges of Bengal tiger (*Panthera tigris tigris L.*) in Pench Tiger Reserve, Madhya Pradesh, Central India | Home range | (Majumder et al., 2012) |
| 24 | Sloth Bear | *Melursus ursinus* | The sloth bear activity and movement in highly fragmented and disturbed habitat in Central India | Habitat use, Activity pattern | (Bargali et al., 2012) |
| 25 | Asiatic Lion | *Panthera leo* | Demographic parameters of endangered Asiatic lions (*Panthera leo persica*) in Gir Forests, India | Mortality | (Banerjee & Jhala, 2012) |
| 26 | Tiger | *Panthera tigris* | Genetic evidence of tiger population structure and migration within an isolated and fragmented landscape in northwest India | Connectivity, migration | (Reddy et al., 2012) |
| 27 | Elephant | *Elephas maximus* | Usual populations, unusual individuals: Insights into the behavior and management of Asian elephants in fragmented landscapes | Behaviour | (Srinivasaiah et al., 2012) |
| 28 | Gaur | *Bos gaurus* | Home range, habitat use and food habits of re-introduced gaur (*Bos gaurus gaurus*) in Bandhavgarh Tiger Reserve, Central | Home range, habitat use of reintroduced animals | (Sankar et al., 2013a) |
| 29 | Asiatic Black Bear | *Ursus thibetanus* | Ecology of Asiatic black bear (*Ursus thibetanus*) in Dachigam National Park, Kashmir, India | Home range, habitat use, movement patterns | (Sambandam, Sharma & Charoo, 2013) |
| 30 | Tiger | *Panthera tigris* | Interbirth interval and litter size of free-ranging Bengal tiger (*Panthera tigris tigris*) in dry tropical deciduous forests of India | Continuous monitoring, interbirth interval and litter size | (Singh et al., 2013) |
| 31 | Leopard | *Panhera pardus* | Ecology of leopard (*Panthera pardus*) in Sariska Tiger Reserve, Rajasthan | Home range and habitat use | (Sankar et al., 2013b) |
| 32 | Smooth-Coated Otter | *Lutrogale perspicillata* | Activity pattern, behavioural activity and interspecific interaction of smooth-coated otter (*Lutrogale perspicillata*) in National Chambal Sanctuary, India. | Activity pattern, habitat use | (Hussain, 2013) |
| 33 | Tiger | *Panthera tigris* | Connectivity of Tiger (*Panthera tigris*) Populations in the Human-Influenced Forest Mosaic of Central India | Connectivity, dispersal | (Joshi et al., 2013) |
| 34 | Leopard | *Panthera pardus* | Home range and resource selection of 'problem' leopards trans-located to forested habitat | Home range | (Mondal et al., 2013) |
| 35 | Tiger | *Panthera tigris* | Philopatry and Dispersal Patterns in Tiger (*Panthera tigris*) | Dispersal | (Gour et al., 2013) |
| 36 | Tiger | *Panthera tigris* | Reintroduction of Tigers in Sariska Tiger Reserve, Rajasthan | Reintroduction, Home range | (Sankar et al., 2013c) |
| 37 | Leopard | *Panthera pardus* | Adaptable Neighbours: Movement Patterns of GPS-Collared Leopards in Human Dominated Landscapes in India | Home range, Movement patterns | (Odden et al., 2014) |
| 38 | Dhole | *Cuon alpinus* | Dhole telemetry studies in Pench Tiger Reserve, Central India | Home range, Movement pattern | (Acharya, Johnsingh & Sankar, 2014) |
| 39 | Tiger | *Panthera tigris* | Movement and activity pattern of a collared tigress in a human-dominated landscape in central India | Home range | (Athreya et al., 2014) |
| 40 | Sambar | *Rusa unicolor* | Ranging pattern and habitat use of Sambar (*Rusa unicolor*) in Sariska Tiger Reserve, Rajasthan, Western India | Home range, Habitat use | (Chatterjee et al., 2014) |
| 41 | One-horned rhino | *Rhinoceros unicornis* | Rehabilitation of greater one-horned rhinoceros calves in Manas National Park, a World Heritage Site in India | Home range, habitat use of reintroduced animals | (Barman et al., 2014) |
| 42 | Lion-tailed Macaque | *Macaca silenus* | Food resources, distribution and seasonal variations in ranging in lion-tailed macaques, *Macaca silenus* in the Western Ghats, India | Home range | (Erinjery, Kavana & Singh, 2015) |
| 43 | Tiger | *Panthera tigris* | Glucocorticoid Stress Responses of Reintroduced Tigers in Relation to Anthropogenic Disturbance in Sariska Tiger Reserve in India | Stress response to anthropogenic disturbances | (Bhattacharjee et al., 2015) |
| 44 | Pygmy Hog | *Porcula salvania* | Radio tracking pygmy hogs – trials of transmitter attachment methods | Transmitter performance | (Narayan & Deka, 2015) |
| 45 | Elephant | *Elephas maximus* | In the elephant's seed shadow: The prospects of domestic bovids as replacement dispersers of three tropical Asian trees | Seed dispersal | (Sekar, Lee & Sukumar, 2015) |
|  | Buffalo | *Bubalus bubalis* |  |  |  |
|  | Cattle | *Bos primigenius* |  |  |  |
| 46 | Asiatic black bear | *Ursus thibetanus* | Conflict Bear Translocation: Investigating Population Genetics and Fate of Bear Translocation in Dachigam National Park, Jammu and Kashmir, India | Movement of translocated bear | (Mukesh et al., 2015) |
| 47 | Lion-tailed Macaque | *Macaca silenus* | Ranging Behavior and Resource Use by Lion-Tailed Macaques (*Macaca silenus*) in Selectively Logged Forests | Home range | (Santhosh et al., 2015) |
| 48 | Western hoolock gibbon | *Hoolock hoolock* | Incorporating movement behavior into conservation prioritization in fragmented landscapes: An example of western hoolock gibbons in Garo Hills, India | Habitat connectivity, home range, habitat use | (Vasudev & Fletcher, 2015) |
| 49 | Rhesus Macaque | *Macaca mulatta* | Primates, Provisioning and Plants: Impacts of Human Cultural Behaviours on Primate Ecological Functions | Day range in provisioned animals | (Sengupta, McConkey & Radhakrishna, 2015) |
| 50 | Tiger | *Panthera tigris* | Multi-scale prediction of landscape resistance for tiger dispersal dispersal in central India | Habitat use, movement patterns | (Krishnamurthy et al., 2016) |
| 51 | Tiger | *Panthera tigris* | Ranging, Activity and Habitat Use by Tigers in the Mangrove Forests of the Sundarban | Home range, Habitat use, activity | (Naha et al., 2016) |
| 52 | Tiger | *Panthera tigris* | Size matters: Scale mismatch between space use patterns of tigers and protected protected area size in a Tropical Dry Forest | Home range | (Singh et al., 2016) |
| 53 | Tiger | *Panthera tigris* | Significance of mate selection and adult sex ratio in tiger reintroduction/reinforcement programs | Mate selection | (Anuradha Reddy et al., 2016) |
| 54 | Tiger | *Panthera tigris* | Movement and home range characteristics of reintroduced tiger (*Panthera tigris*) population in Panna Tiger Reserve, central India | Home range, movement | (Sarkar et al., 2016) |
| 55 | Eastern Hoolock Gibbon | *Hoolock leuconedys* | The day range and home range of the Eastern Hoolock Gibbon *Hoolock leuconedys* (Mammalia: Primates: Hylobatidae) in Lower Dibang Valley District in Arunachal Pradesh, India | Home range | (Sarma & Kumar, 2016) |
| 56 | Gaur | *Bos gaurus* | Diurnal Activity Budgets and Activity Patterns of a Reintroduced Gaur Population in Bandhavgarh Tiger Reserve, Madhya Pradesh, Central India | Activity budgets and pattern | (Manjrekar et al., 2017) |
| 57 | Tiger | *Panthera tigris* | Demography of a small, isolated tiger (Panthera tigris tigris) population in a semi- arid region of western India | Demography | (Sadhu et al., 2017) |
| 58 | Elephant | *Elephas maximus* | Functional nonredundancy of elephants in a disturbed tropical forest | Seed dispersal | (Sekar, Lee & Sukumar, 2017) |
|  | Buffalo | *Bubalus bubalis* |  |  |  |
|  | Cattle | *Bos primigenius* |  |  |  |
|  | Rhesus Macaque | *Macaca mulatta* |  |  |  |
| 59 | Tiger | *Panthera tigris* | Long-term monitoring of a Bengal tiger (*Panthera tigris tigris*) population in a human-dominated landscape of Central India | Population monitoring | (Majumder et al., 2017) |
| 60 | Dhole | *Cuon alpinus* | Field report Home range size of the dhole estimated from camera- trap surveys | Home-range | (Srivathsa, Kumar & Karanth, 2017) |
| 61 | One-horned rhino | *Rhinoceros unicornis* | Behaviour of post released translocated greater one-horned rhinoceros (*Rhinoceros unicornis*) at Manas National Park, Assam, India | Activity of translocated animal | (Dutta et al., 2017) |
| 62 | Dog-faced Fruit Bat | *Cynopterus brachyots* | Foraging and roosting ecology of the Lesser Dog-faced Fruit Bat *Cynopterus brachyotis* (Mammalia: Chiroptera: Pteropodidae) in southern India | Roosting ecology, Foraging | (Karuppudurai & Sripathi, 2018) |
| 63 | Tiger | *Panthera tigris* | New insights into the factors influencing in the human-dominated buffer zone of Panna reintroduced Bengal tigers (*Panthera tigris tigris*) movements and spatial distribution of Tiger Reserve, India | Factors affecting tiger presence | (Kolipaka et al., 2018) |
| 64 | Elephant | *Elephas maximus* | Ranging and Spacing Behaviour of Asian Elephant (*Elephas maximus Linnaeus*) in the Tropical Forests of Southern India | Home-range, Habitat use | (Venkataraman et al., 2005) |
| 65 | Indian Foxes | *Vulpes bengalensis* | Right on track? Performance of satellite telemetry in terrestrial wildlife research | Satellite telemetry performance | (Hofman et al., 2019) |
|  | Golden Jackal | *Canis aureus* |  |  |  |
|  | Jungle Cat | *Felis chaus* |  |  |  |
| 66 | Leopard | *Panthera pardus* | Ecology of Leopard (*Panthera pardus fusca Meyer*) in Dry Tropical Forests of Gir National Park and Sanctuary, Gujarat, India | Home range | (Zehra, Chaudhary & Khan, 2010) |
| 67 | Leopard | *Panthera pardus* | A Camera-Trap Home-Range Analysis of the Indian Leopard (*Panthera pardus fusca*) in Jaipur, India | Home range | (Kumbhojkar et al., 2020) |
| 68 | Indian Foxes | *Vulpes bengalensis* | Effects of body size on estimation of mammalian area requirements | Body size and Home range relationship | (Noonan et al., 2020) |
|  | Golden Jackal | *Canis aureus* |  |  |  |
|  | Jungle Cat | *Felis chaus* |  |  |  |
| 69 | Tiger | *Panthera tigris* | Acquisition of vacated home ranges by tigers | Home range | (Singh et al., 2020) |
| 70 | Rhesus Macaque | *Mucaca mulatta* | The Role of Landscape Structure in Primate Crop Feeding: Insights from Rhesus Macaques (*Macaca mulatta*) in Northern India | Habitat selection, Home range | (Anand, Vaidyanathan & Radhakrishna, 2021) |
| 71 | Tiger | *Panthera tigris* | Tigers and leopards coexist despite similarities in space use and habitat selection | Habitat selection | (Kumar, Karanth & Jathanna, 2020; Singh et al., 2021) |
|  | Leopard | *Panthera pardus* |  |  |  |
| 72 | Tiger | *Panthera tigris* | Philopatric and natal dispersal of tigers in a semi-arid habitat, western India | Dispersal | (Singh et al., 2021) |
| 73 | Leopard | *Panthera pardus* | Movement behavior of a solitary large carnivore within a hotspot of human‑wildlife conflicts in India | Home range, Movement pattern, Resource selection | (Naha et al., 2021) |
| 74 | Tiger | *Panthera tigris* | Not a cakewalk: Insights into movement of large carnivores in human-dominated landscapes in India | Movement pattern | (Habib et al., 2021) |
|  | Leopard | *Panthera pardus* |  |  |  |
|  | Dhole | *Cuon alpinus* |  |  |  |
|  | Indian Wolf | *Canis lupus pallipes* |  |  |  |
| 75 | Tiger | *Panthera tigris* | The spacing pattern of reintroduced tigers in human-dominated Sariska Tiger Reserve | Home Range | (Bhardwaj et al., 2021) |
| 76 | Sloth Bear | *Melursus ursinus* | Relocation of a GPS collared Sloth Bear *Melursus ursinus* (Mammalia: Carnivora) in Karnataka, India | Monitoring movement of relocated animal | (Arun et al., 2021) |
| 77 | Lesser false vampire bat | *Megaderma spasma* | Ecological drivers of selection for remnant forest habitats by an insectivorous bat in a tropical, human-modified landscape | Habitat use | (Prakash et al., 2021) |
| 78 | Golden Jackal | *Canis aureus* | Habitat specificity drives differences in space use patterns of multiple mesocarnivores in an agro-ecosystem | Home range, Habitat selection | (Katna et al., 2021) |
|  | Jungle Cat | *Felis chaus* |  |  |  |
|  | Indian Foxes | *Vulpes bengalensis* |  |  |  |
| 79 | Tiger | *Panthera tigris* | Long-distance dispersal and home range establishment by a female sub-adult tiger (*Panthera tigris*) in the Panna landscape, central India | Dispersal distance, Home range | (Sarkar et al., 2021) |
| 80 | Swamp Deer | *Rucervus duvaucelii* | Waning grasslands: a quantitative temporal evaluation of the grassland habitats across human-dominated upper Gangetic Plains, north India | Home range and habitat selection | (Paul et al., 2021) |
| 81 | Leopard | *Pathera pardus* | Home range variation in leopards living across the human density gradient | Home range | (Snider et al., 2021) |
| 82 | Human | *Homo sapiens* | Human movement influenced by perceived risk of wildlife encounters at fine scales: Evidence from central India | Human movement, Wildlife conflict | (Read et al., 2021) |

***Table S3.*** *News articles and video links of monitoring and tracking studies (either proposed or ongoing) in various parts of India.*

| **No.** | **News Article** | **Species** | **State** | **Source** |
| --- | --- | --- | --- | --- |
| 1. | Why elephants thriving in Karnataka’s coffee estates isn’t good news | Elephant | Karnataka | [**link**](https://scroll.in/article/1027161/why-elephants-thriving-in-karnataka-s-coffee-estates-isn-t-good-news) |
| 2. | i. Radio collar use to track elephant movement in Sonitpur district;  ii. Explained: Can elephant collaring help manage human-elephant conflict in Assam? | Elephant | Assam | [**link**](https://www.sentinelassam.com/north-east-india-news/assam-news/radio-collar-use-to-track-elephant-movement-in-sonitpur-district-563632)**,** [**link**](https://indianexpress.com/article/explained/assam-human-elephant-conflict-radio-collars-7636680/) |
| 3. | Uttarakhand to tag wild elephants with radio collars | Elephant | Uttarakhand | [**link**](https://indianexpress.com/article/india/uttarakhand-to-tag-wild-elephants-with-radio-collars-6113548/) |
| 4. | Odisha readies radio collars for 7 elephants: Why experts don't like that | Elephant | Odisha | [**link**](https://www.downtoearth.org.in/news/wildlife-biodiversity/odisha-readies-radio-collars-for-7-elephants-why-experts-don-t-like-that-79113) |
| 5. | GPS collars to track elephant herds | Elephant | West Bengal | [**link**](https://www.telegraphindia.com/west-bengal/gps-collars-to-track-elephant-herds/cid/1689321) |
| 6. | Tripura to radio collar elephants to minimize human-jumbo conflict | Elephant | Tripura | [**link**](https://indianexpress.com/article/north-east-india/tripura/tripura-to-radio-collar-elephants-to-minimize-human-jumbo-conflict/) |
| 7. | Mumbai's radio-collared leopards send interesting data | Leopard | Maharashtra | [**link**](http://www.dailypioneer.com/2021/india/mumbai-s-radio-collared-leopards-send-interesting-data.html) |
| 8. | Indian striped hyena rescued as cub, raised in captivity, released into the wild | Striped Hyena | Maharashtra | [**link**](https://timesofindia.indiatimes.com/city/pune/indian-striped-hyena-rescued-as-cub-raised-in-captivity-released-into-the-wild/articleshow/91569997.cms?from=mdr) |
| 9. | Madhya Pradesh To Radio Collar Leopard, Hyenas To Understand Their Behaviour With Namibian Cheetahs | Leopard, Striped Hyena and Cheetah | Madhya Pradesh | [**link**](https://www.outlookindia.com/national/madhya-pradesh-to-radio-collar-leopard-hyenas-to-understand-their-behaviour-with-namibian-cheetahs-news-214181) |
| 10 | Hidden in Plain Sight | Striped Hyena | Maharashtra, Karnataka | [**link**](https://www.youtube.com/watch?v=vduaDm5Rnjk&ab_channel=ATREEBangalore) |
| 11. | Tracking the Incredible Journey of the Amur Falcon | Amur Falcon | Nagaland, Manipur | [**link**](https://www.conservationindia.org/articles/tracking-the-incredible-journey-of-the-amur-falcon)**,**  [**link**](https://nenow.in/north-east-news/manipur-satellite-tagged-amur-falcon-tamenglong-reaches-somalia-five-days-flight.html) |
| 12. | WII tracks return migration of Common Crane from Kazakhstan | Common Crane | Gujarat | [**link**](https://www.thehansindia.com/news/national/wii-tracks-return-migration-of-common-crane-from-kazakhstan-651243) |
| 13. | The Forest Owlet | Forest owlet | Madhya Pradesh | [**link**](https://www.sanctuarynaturefoundation.org/article/the-forest-owlet) |
| 14. | Great Indian Bustard: WII plans to carry out surveys, use GPS to track GIB population | Great Indian Bustard | Rajasthan, Gujarat | [**link**](https://energy.economictimes.indiatimes.com/news/renewable/great-indian-bustard-wii-plans-to-carry-out-surveys-use-gps-to-track-gib-population/80360543) |
| 15. | In a first, BNHS to use cellular tech for studying bird migration | Flamingo, Ibis, Curlews | Maharashtra | [**link**](https://indianexpress.com/article/cities/mumbai/in-a-first-bnhs-to-use-cellular-tech-for-studying-bird-migration-6259554/) |
| 16. | Radio-transmitters tracking a canopy katydid | Katydid (*Onomarchus uninotatus)* | Karnataka | [**link**](https://sites.google.com/view/rohinibalakrishnanlab/gallery/kudremukh-crickets) |
| 17. | Flamingo Tracking Project | Flamingo | Gujarat | [**link**](https://www.youtube.com/watch?v=5tV_F4OYumU&ab_channel=ManishRManick) |
| 18. | Movements of a Eurasian crane tagged in Western Gujarat, India | Eurasian Crane | Gujarat | [**link**](https://www.researchgate.net/publication/358549925_Movements_of_a_Eurasian_Crane_Common_Crane_tagged_in_Western_Gujarat_India) |
| 19. | Mandya: GPS tagging to help know lives of Pelicans better | Spot-billed Pelican | Karnataka | [**link**](https://timesofindia.indiatimes.com/city/hubballi/gps-tagging-to-help-know-lives-of-pelicans-better/articleshow/85014959.cms) |
| 20. | Vulture, rescued during cyclone Ockhi, ready to spread wings in Rajasthan | Cinereous Vulture | Tamil Nadu | [**link**](https://www.thehindu.com/news/cities/Coimbatore/vulture-rescued-during-cyclone-ockhi-ready-to-spread-wings-in-rajasthan/article65863076.ece) |
| 21. | Turtle travels: Tracking olive ridleys in the Arabian Sea | Olive ridley turtle | Maharashtra | [**link**](https://www.hindustantimes.com/lifestyle/art-culture/turtle-travels-tracking-olive-ridleys-in-the-arabian-sea-101662127386365.html) |
| 22. | As vulture count dips, study begins in Pong reservoir | White-rumped Vultures | Himachal Pradesh | [**link**](https://www.tribuneindia.com/news/himachal/as-vulture-count-dips-study-begins-in-pong-reservoir-341721) |
| 23. | Cuckoos to be tracked by WII, IIRS in 1st attempt to decode link between ‘rain bird’, monsoon & climate change | Jacobin Cuckoo | Uttarakhand | [**link**](https://timesofindia.indiatimes.com/city/dehradun/cuckoos-to-be-tracked-by-wii-iirs-in-1st-attempt-to-decode-link-between-rain-bird-monsoon-climate-change/articleshow/76966093.cms) |
| 24. | Geotagging to monitor vultures in MP’s Panna Tiger Reserve | Indian vultures, Himalayan griffon Vultures, Eurasian Griffon Vultures & King Vultures | Madhya Pradesh | [**link**](https://india.mongabay.com/2022/04/geotagging-to-monitor-vultures-in-mps-panna-tiger-reserve/#:~:text=Around%2025%20vultures%20in%20Panna,raptors%20in%20the%20protected%20area) |
| 25. | Gujarat Forest Dept Marks Endangered Lesser Florican Birds with Satellite Tags | Lesser Florican | Gujarat | [**link**](https://www.newshoundindia.foundation/blog-details.asp?id=333) |
| 26. | Gujarat reintroduces Indian grey hornbills in Gir in second attempt after 1980 | Indian Grey Hornbill | Gujarat | [**link**](https://indianexpress.com/article/cities/rajkot/gujarat-reintroduces-indian-grey-hornbills-in-gir-after-eight-decades-7794311/) |
| 27. | Gujarat Forest Department has tagged two Demoiselle Cranes & two Common Cranes with solar-powered transmitters | Demoiselle Cranes, Common Cranes | Gujarat | [**link**](https://m.facebook.com/watch/?v=1118359712039565&paipv=0&eav=Afb9kvpytJ4qUjG0zqeh8gw_0ScF6MCKQFqaBtXYOMKtiR1s5oOM304H7F7AiTNNe0E&_rdr) |

**References**

Acharya B, Johnsingh AJT, Sankar K. 2014. Dhole telemetry studies in Pench Tiger Reserve, Central India. *Telemetry in Wildlife Science* 13:68–78.

Anand S, Vaidyanathan S, Radhakrishna S. 2021. The Role of Landscape Structure in Primate Crop Feeding: Insights from Rhesus Macaques (Macaca mulatta) in Northern India. *International Journal of Primatology* 42:764–780. DOI: 10.1007/s10764-021-00238-y.

Anuradha Reddy P, Ramesh K, Shekhar Sarkar M, Srivastava A, Bhavanishankar M, Shivaji S. 2016. Significance of mate selection and adult sex ratio in tiger reintroduction/reinforcement programs. *Journal of Zoology* 299:132–141. DOI: 10.1111/jzo.12331.

Arrowsmith LM, Paidi CK, Bloch FH, John S, Choudhury BC, Kaul R, Sequeira AMM, Pattiaratchi CB, Meekan MG. 2021. First Insights Into the Horizontal Movements of Whale Sharks (Rhincodon typus) in the Northern Arabian Sea. *Frontiers in Marine Science* 8. DOI: 10.3389/fmars.2021.682730.

Arun AS, Swaminathan S, Pannerselvam Y, Sharp TR, Stephens SR, Satyanarayan K, Seshamani G. 2021. Relocation of a GPS collared conflict Sloth Bear Melursus ursinus (Mammalia: Carnivora) in Karnataka, India. *Journal of Threatened Taxa* 13:17856–17864. DOI: 10.11609/jott.5947.13.3.17856-17864.

Athreya V, Navya R, Punjabi GA, Linnell JDC, Odden M, Khetarpal S, Karanth KU. 2014. Movement and activity pattern of a collared tigress in a human-dominated landscape in central India Methods Study area. *Tropical Conservation Science* 7:75–86. DOI: 10.1177/194008291400700111.

Banerjee K, Jhala Y V. 2012. Demographic parameters of endangered Asiatic lions ( Panthera leo persica ) in Gir Forests , India. *Journal of Mammalogy* 93:1420–1430. DOI: 10.1644/11-MAMM-A-231.1.

Bargali HS, Foundation TC, Akhtar N, Zoo R. 2012. The sloth bear activity and movement in highly fragmented and disturbed habitat in Central India. *World Journal of Zoology* 7:312–319. DOI: 10.5829/idosi.wjz.2012.7.4.64180.

Barman R, Choudhury B, Ashraf N, Menon V. 2014. Rehabilitation of greater one-horned rhinoceros calves in Manas National Park, a World Heritage Site in India. *Pachyderm* 55:78–88.

Barve S, Bhaisare D, Giri a, Shankar PG, Whitaker R, Goode M. 2013. A preliminary study on translocation of “rescued” King Cobras (Ophiophagus hannah). *Hamadryad* 36:80–86.

Behera S, Tripathy B, Choudhury BC, Sivakumar K. 2018. Movements of Olive Ridley Turtles (Lepidochelys olivacea) in the Bay of Bengal, India, Determined via Satellite Telemetry. *Chelonian Conservation and Biology* 17:44–53. DOI: 10.2744/CCB-1245.1.

Bhardwaj S, Selvi G, Agasti S, Kari B, Singh H, Kumar A, Gupta R, Reddy G. 2021. The spacing pattern of reintroduced tigers in human-dominated Sariska Tiger Reserve. *Journal of Wildlife and Biodiversity* 5:1–14.

Bhattacharjee S, Kumar V, Chandrasekhar M, Malviya M, Ganswindt A, Ramesh K, Sankar K, Umapathy G. 2015. Glucocorticoid Stress Responses of Reintroduced Tigers in Relation to Anthropogenic Disturbance in Sariska Tiger Reserve in India. *PLoS ONE* 10:e0127626. DOI: 10.1371/journal.pone.0127626.

Chatterjee D, Sankar K, Qureshi Q, Malik PK, Nigam P, Dun D. 2014. Ranging pattern and habitat use of Sambar ( Rusa unicolor ) in Sariska Tiger Reserve ,. *DSG Newsletter* 26:60–71.

Deepak V, Noon BR, Vasudevan K. 2016. Fine Scale Habitat Selection in Travancore Tortoises (Indotestudo travancorica) in the Anamalai Hills, Western Ghats. *Journal of Herpetology* 50:278–283. DOI: 10.1670/14-024.

Dutta DK, Sharma A, Mahanta R, Swargowari A. 2017. Behaviour of post released translocated greater one-horned rhinoceros (Rhinoceros unicornis) at Manas National Park, Assam, India. *Pachyderm* 2017:58–66.

Erinjery JJ, Kavana TS, Singh M. 2015. Food resources, distribution and seasonal variations in ranging in lion-tailed macaques, Macaca silenus in the Western Ghats, India. *Primates* 56:45–54. DOI: 10.1007/s10329-014-0447-x.

Glaudas X. 2021. Proximity between humans and a highly medically significant snake, Russell’s viper, in a tropical rural community. *Ecological Applications* 31:1–8. DOI: 10.1002/eap.2330.

Gour DS, Bhagavatula J, Bhavanishankar M, Reddy PA, Gupta JA, Sarkar MS, Hussain SM, Harika S, Gulia R, Shivaji S. 2013. Philopatry and Dispersal Patterns in Tiger (Panthera tigris). *PLoS ONE* 8:14–17. DOI: 10.1371/journal.pone.0066956.

Habib B, Ghaskadbi P, Khan S, Hussain Z, Nigam P. 2021. Not a cakewalk: Insights into movement of large carnivores in human-dominated landscapes in India. *Ecology and Evolution* 11:1653–1666. DOI: 10.1002/ece3.7156.

Hawkes LA, Balachandran S, Batbayar N, Butler PJ, Frappell PB, Milsom WK, Tseveenmyadag N, Newman SH, Scott GR, Sathiyaselvam P, Takekawa JY, Wikelski M, Bishop CM. 2011. The trans-Himalayan flights of bar-headed geese (Anser indicus). *Proceedings of the National Academy of Sciences of the United States of America* 108:9516–9519. DOI: 10.1073/pnas.1017295108.

Hofman MPG, Hayward MW, Heim M, Marchand P, Rolandsen CM, Balkenhol N. 2019. Right on track? Performance of satellite telemetry in terrestrial wildlife research. *PLoS ONE* 14:e0216223.

Hussain SA. 2013. Activity pattern, behavioural activity and interspecific interaction of smooth-coated otter (Lutrogale perspicillata) in National Chambal Sanctuary, India. *IUCN Otter Specialist Group Bulletin* 30:5–17.

Jha AK. 2011. *Release and Reintroduction of Captive-Bred Red Pandas into Singalila National Park, Darjeeling, India*. DOI: 10.1016/B978-1-4377-7813-7.00025-2.

Jha RRS, Thakuri JJ, Rahmani AR, Dhakal M, Khongsai N, Pradhan NMB, Shinde N, Chauhan BK, Talegaonkar RK, Barber IP, Buchanan GM, Galligan TH, Donald PF. 2018. Distribution, movements, and survival of the critically endangered Bengal Florican Houbaropsis bengalensis in India and Nepal. *Journal of Ornithology* 159:851–866. DOI: 10.1007/s10336-018-1552-1.

Joshi A, Vaidyanathan S, Mondol S, Edgaonkar A, Ramakrishnan U. 2013. Connectivity of Tiger ( Panthera tigris ) Populations in the Human-Influenced Forest Mosaic of Central India. *PLoS ONE* 8:e77980. DOI: 10.1371/journal.pone.0077980.

Karuppudurai T, Sripathi K. 2018. Foraging and roosting ecology of the Lesser dog-faced fruit bat Cynopterus brachyotis (Mammalia: Chiroptera: Pteropodidae) in Southern Indian. *Journal of Threatened Taxa* 10:12163–12172.

Katna A, Kulkarni A, Thaker M, Vanak AT. 2021. Habitat specificity drives differences in space use patterns of multiple mesocarnivores in an agro-ecosystem. *Journal of Zoology*.

Kolipaka SS, Tamis WLM, Van’t Zelfde M, Persoon GA, de Iongh HH. 2018. New insights into the factors influencing movements and spatial distribution of reintroduced Bengal tigers (Panthera tigris tigris) in the human-dominated buffer zone of Panna Tiger Reserve, India. *Mammalia* 82:207–217. DOI: 10.1515/mammalia-2016-0126.

Krishnamurthy R, Cushman SA, Sarkar MS, Malviya M, Naveen M, Johnson JA, Sen S. 2016. Multi-scale prediction of landscape resistance for tiger dispersal in central India. *Landscape Ecology* 31:1355–1368. DOI: 10.1007/s10980-016-0363-0.

Kumar N, Gupta U, Jhala Y V., Qureshi Q, Gosler AG, Sergio F. 2020. GPS-telemetry unveils the regular high-elevation crossing of the Himalayas by a migratory raptor: implications for definition of a “Central Asian Flyway.” *Scientific Reports* 10:1–9. DOI: 10.1038/s41598-020-72970-z.

Kumar A, Karanth KU, Jathanna D. 2020. Tigers and leopards coexist despite similarities in space use and habitat selection. *Cat News*:20–23.

Kumbhojkar S, Yosef R, Mehta A, Rakholia S. 2020. A Camera-Trap Home-Range Analysis of the Indian Leopard (Panthera pardus fusca) in Jaipur, India. *Animals* 10:1–22.

Kuppusamy S, Jhala Y V, Bhardwaj GS, Mohan A. 2017. *A Study on Ecology and Migration of the Lesser Florican (Sypheotides indica) in Western India Using Satellite Tracking Techniques*. DOI: 10.13140/RG.2.2.24926.18248.

Mahar N, Habib B, Shawl T, Gopi GV, Suhail I, Takpa J, Hussain SA. 2015. Tracking the movement pattern of Bar-headed Goose anser indicus captured from the Gharana conservation reserve, India. *Journal of the Bombay Natural History Society* 112:14–22. DOI: 10.17087/jbnhs/2015/v112i1/92194.

Majumder A, Basu S, Sankar1 K, Qureshi1 Q, Jhala YV, Nigam P, Gopal R. 2012. Home ranges of Bengal tiger (Panthera tigris tigris L.) in Pench Tiger Reserve , Madhya Pradesh, Central India. *Wildlife Biology in Practice* 8:36–49. DOI: 10.2461/wbp.2012.8.4.

Majumder A, Qureshi Q, Sankar K, Kumar A. 2017. Long-term monitoring of a Bengal tiger (Panthera tigris tigris) population in a human-dominated landscape of Central India. *European Journal of Wildlife Research* 63:1–11. DOI: 10.1007/s10344-016-1070-5.

Manjrekar MP, Navaneethan B, Nigam P, Qureshi Q, Sankar K. 2017. Diurnal activity budgets and activity patterns of a reintroduced gaur population in Bandhavgarh Tiger Reserve, Madhya Pradesh, Central India. *Asian Journal of Conservation Biology* 6:31–37.

Mohit K, Kumar S, Rahmani AR, Khan JA, Belal SM, Khan AM. 2011. Satellite tracking of Bar-headed Geese Anser indicus wintering in Uttar Pradesh, India. *Journal of the Bombay Natural History Society* 108:79–94.

Mondal K, Bhattacharjee S, Gupta S, Sankar K, Qureshi Q. 2013. Home range and resource selection of “problem” leopards trans-located to forested habitat. *Current Science* 105:338–345.

Mondal I, Kumar RS, Habib B, Talukdar G. 2016. Modelling fine scale movement corridors for the Tricarinate Hill turtle. *International Archives of the Photogrammetry, Remote Sensing and Spatial Information Sciences* 41:719–725. DOI: 10.5194/isprsarchives-XLI-B8-719-2016.

Mukesh, Sharma LK, Charoo SA, Sathyakumar S. 2015. Conflict bear translocation: Investigating population genetics and fate of bear translocation in Dachigam National Park, Jammu and Kashmir, India. *PLoS ONE* 10:1–17. DOI: 10.1371/journal.pone.0132005.

Naha D, Dash SK, Kupferman C, Beasley JC, Sathyakumar S. 2021. Movement behavior of a solitary large carnivore within a hotspot of human-wildlife conflicts in India. *Scientific Reports* 11:1–14. DOI: 10.1038/s41598-021-83262-5.

Naha D, Jhala Y, Qureshi Q, Roy M, Sankar K, Gopal R. 2016. Ranging, Activity and Habitat Use by Tigers in the Mangrove Forests of the Sundarban. *PLoS ONE* 11:e0152119. DOI: 10.1371/journal.pone.0152119.

Namgail T, Takekawa JY, Bala- S, Sathiyaselvam P, Mundkur T, Scott H. 2014. Space use of wintering waterbirds in India : Influence of trophic ecology on home-range size. *Current Zoology* 60:616–621.

Namgail T, Takekawa JY, Sivananinthaperumal B, Sathiyaselvam P, Areendran G, Mundkur T, Mccracken T, Newman S. 2011. Ruddy Shelduck Tadorna ferruginea home range and habitat use during the non-breeding season in Assam, India. *Wildfowl* 61:182–193.

Naniwadekar R, Rathore A, Shukla U, Chaplod S, Datta A. 2019. How far do Asian forest hornbills disperse seeds? *Acta Oecologica* 101:103482. DOI: 10.1016/j.actao.2019.103482.

Naniwadekar R, Rathore A, Shukla U, Datta A. 2021. Roost site use by Great (Buceros bicornis) and Wreathed (Rhyticeros undulatus) Hornbill and its implications for seed dispersal. *Biotropica*:1–5. DOI: 10.1111/btp.13039.

Narayan G, Deka PJ. 2015. Radio tracking pygmy hogs – trials of transmitter attachment methods. *Solitaire* 26:19–26.

Noonan MJ, Fleming CH, Tucker MA, Kays R, Harrison A, Crofoot MC, Abrahms B, Alberts SC, Ali AH, Altmann J, Antunes PC, Attias N, Belant JL, Jr DEB, Bidner LR, Blaum N, Boone RB, Caillaud D, Paula RC de, Torre JA de, Dekker J, Deperno CS, Farhadinia M, Fennessy J, Fichtel C, Fischer C, Ford A, Goheen JR, Havmøller RW, Hirsch BT, Kaneko Y, Kappeler P, Katna A, Kauffman M, Koch F, Kulkarni A, Lapoint S, Leimgruber P, Macdonald DW, Markham AC, Mcmahon L, Mertes K, Moorman CE, Morato RG, Moßbrucker AM, Mourão G, Connor DO, Oliveira-santos LGR, Pastorini J, Patterson BD, Rachlow J, Ranglack DH, Reid N, Scantlebury DM, Scott DM, Selva N, Sergiel A, Songer M, Songsasen N, Stabach JA, Stacy-dawes J, Swingen MB, Wilson JW, Yamazaki K, Yarnell RW, Zieba F, Zwijacz-kozica T, Fagan WF, Mueller T, Calabrese JM. 2020. Effects of body size on estimation of mammalian area requirements. 34:1017–1028. DOI: 10.1111/cobi.13495.

Odden M, Athreya V, Rattan S, Linnell JDC. 2014. Adaptable Neighbours: Movement Patterns of GPS-Collared Leopards in Human Dominated Landscapes in India. *PLoS ONE* 9:e112044. DOI: 10.1371/journal.pone.0112044.

Paul S, Saha S, Nigam P, Ali SZ, Page N, Khan, A. S. \, Mondol S. 2021. Waning grasslands: a quantitative temporal evaluation of the grassland habitats across human-dominated upper Gangetic Plains, north India. *BioRxiv.* DOI: https://doi.org/10.1101/2021.10.10.463811.

Prakash H, Saha K, Sahu S, Balakrishnan R. 2021. Ecological drivers of selection for remnant forest habitats by an insectivorous bat in a tropical , human-modified landscape. *Forest Ecology and Management* 496:119451. DOI: 10.1016/j.foreco.2021.119451.

Ramaswami G, Kaushik M, Prasad S, Sukumar R, Westcott D. 2016. Dispersal by generalist frugivores affects management of an invasive plant. 0:1–7.

Rana S, Kalsi RS, Burra MR. 2012. Home range comparison of male and female Grey francolin (Francolinus pondicerianus) using radiotelemetry. *Records of the Zoological Survey of India* 112:13–18.

Rao C, Talukdar G, Choudhury BC, Shankar PG, Whitaker RE, Goode M. 2013. Habitat use of King Cobra (Ophiophagus hannah) in a heterogeneous landscape matrix in the tropical forests of the Western Ghats, India. *Hamadryad* 36:69–79.

Read DJ, Habib B, Stabach J, Leimgruber P. 2021. Human movement influenced by perceived risk of wildlife encounters at fine scales: Evidence from central India. *Biological Conservation* 254:108945. DOI: 10.1016/j.biocon.2020.108945.

Reddy PA, Gour DS, Bhavanishankar M, Jaggi K, Hussain SM, Harika K, Shivaji S. 2012. Genetic evidence of tiger population structure and migration within an isolated and fragmented landscape in northwest India. *PLoS ONE* 7. DOI: 10.1371/journal.pone.0029827.

Sadhu A, Jayam PPC, Qureshi Q, Shekhawat RS, Sharma S, Jhala YV. 2017. Demography of a small, isolated tiger (Panthera tigris tigris) population in a semi-arid region of western India. *BMC Zoology* 2:1–13. DOI: 10.1186/s40850-017-0025-y.

Sambandam S, Sharma LK, Charoo SA. 2013. *Ecology of Asiatic black bear (Ursus thibetanus) in Dachigam National Park, Kashmir, India*.

Sankar K, Pabla HS, Patil CK, Nigam P, Qureshi Q, Navaneethan B, Manjreakar M, Virkar PS, Mondal K. 2013a. Home range, habitat use and food habits of re-introduced gaur (Bos gaurus gaurus) in Bandhavgarh Tiger Reserve, central India. *Tropical Conservation Science* 6:50–69. DOI: 10.1177/194008291300600108.

Sankar K, Qureshi Q, Jhala V, Mondal K, Gupta S, Chourasia P. 2013b. *Ecology of leopard (Panthera Pardus) in Sariska Tiger Reserve, Rajasthan*.

Sankar K, Qureshi Q, Malik PK, Nigam P, Sinha PR, Mehrotra RN, Gopal R. 2013c. Reintroduction of Tigers in Sariska Tiger Reserve, Rajasthan. In: *Faunal Heritage of Rajasthan, India*. Springer Cham, 157–171.

Santhosh K, Kumara HN, Velankar AD, Sinha A. 2015. Ranging Behavior and Resource Use by Lion-Tailed Macaques (Macaca silenus) in Selectively Logged Forests. *International Journal of Primatology* 36:288–310. DOI: 10.1007/s10764-015-9824-6.

Sarkar MS, Niyogi R, Masih RL, Hazra P, Maiorano L, John R. 2021. Long-distance dispersal and home range establishment by a female sub-adult tiger (Panthera tigris) in the Panna landscape, central India. *European Journal of Wildlife Research* 67:1–7. DOI: 10.1007/s10344-021-01494-2.

Sarkar MS, Ramesh K, Johnson JA, Sen S, Nigam P, Gupta SK. 2016. Movement and home range characteristics of reintroduced tiger ( Panthera tigris ) population in Panna Tiger Reserve , central India. *European Journal of Wildlife Research* 62:537–547. DOI: 10.1007/s10344-016-1026-9.

Sarma K, Kumar A. 2016. The day range and home range of the Eastern Hoolock Gibbon Hoolock leuconedys (Mammalia: Primates: Hylobatidae) in Lower Dibang Valley District in Arunachal Pradesh, India. *Journal of Threatened Taxa* 8:8641–8651. DOI: 10.11609/jott.2739.8.4.8641-8651.

Sekar N, Lee C Lo, Sukumar R. 2015. In the elephant’s seed shadow: The prospects of domestic bovids as replacement dispersers of three tropical Asian trees. *Ecology* 96:2093–2105. DOI: 10.1890/14-1543.1.

Sekar N, Lee C lo, Sukumar R. 2017. Functional nonredundancy of elephants in a disturbed tropical forest. *Conservation Biology* 31:1152–1162. DOI: 10.1111/cobi.12907.

Sengupta A, McConkey KR, Radhakrishna S. 2015. Primates, provisioning and plants: Impacts of human cultural behaviours on primateecological functions. *PLoS ONE* 10:1–13. DOI: 10.1371/journal.pone.0140961.

Singh R, Majumder A, Sankar K, Qureshi Q, Goyal SP, Nigam P. 2013. Interbirth interval and litter size of free-ranging Bengal tiger (Panthera tigris tigris) in dry tropical deciduous forests of India. *European Journal of Wildlife Research* 59:629–636. DOI: 10.1007/s10344-013-0713-z.

Singh R, Pandey P, Qureshi Q, Sankar K, Krausman PR, Goyal SP. 2020. Acquisition of vacated home ranges by tigers. *Current Science* 119:1549–1554. DOI: 10.18520/cs/v119/i9/1549-1554.

Singh R, Pandey P, Qureshi Q, Sankar K, Krausman PR, Goyal SP. 2021. Philopatric and natal dispersal of tigers in a semi-arid habitat, western India. *Journal of Arid Environments* 184:104320. DOI: 10.1016/j.jaridenv.2020.104320.

Singh R, Sharma K, Gogate N, Malik PK, Tamim A. 2016. Size matters: Scale mismatch between space use patterns of tigers and protected area size in a Tropical Dry Forest. *Biological Conservation* 197:146–153. DOI: 10.1016/j.biocon.2016.03.004.

Snider MH, Athreya VR, Balme GA, Bidner LR, Farhadinia MS, Fattebert J, Gompper ME, Gubbi S, Hunter LTB, Isbell LA, Macdonald DW, Odden M, Owen CR, Slotow R, Spalton JA, Stein AB, Steyn V, Vanak AT, Weise FJ, Wilmers CC, Kays R. 2021. Home range variation in leopards living across the human density gradient. *Journal of Mammalogy* 102:1138–1148. DOI: 10.1093/jmammal/gyab068.

Srinivasaiah NM, Anand VD, Vaidyanathan S, Sinha A. 2012. Usual populations, unusual individuals: Insights into the behavior and management of Asian elephants in fragmented landscapes. *PLoS ONE* 7. DOI: 10.1371/journal.pone.0042571.

Srivathsa A, Kumar NS, Karanth KU. 2017. Field report Home range size of the dhole estimated from camera- trap surveys. *Canid Biology and Conservation* 20:1–4.

Swaminathan A, Namboothri N, Shanker K. 2019. Tracking Leatherback Turtles From Little Andaman. *Indian Ocean Turtle Newsletter*:8–10.

Terraube J, Mougeot F, Cornulier T, Verma A, Gavrilov A, Arroyo B. 2012. Broad wintering range and intercontinental migratory divide within a core population of the near-threatened pallid harrier. *Diversity and Distributions* 18:401–409. DOI: 10.1111/j.1472-4642.2011.00830.x.

Vasudev D, Fletcher RJ. 2015. Incorporating movement behavior into conservation prioritization in fragmented landscapes: An example of western hoolock gibbons in Garo Hills, India. *Biological Conservation* 181:124–132. DOI: 10.1016/j.biocon.2014.11.021.

Venkataraman AB, Saandeep R, Baskaran N, Roy M, Madhivanan A, Sukumar R. 2005. Using satellite telemetry to mitigate elephant-human conflict: An experiment in northern West Bengal, India. *Current Science* 88:1827–1831.

Zehra N, Chaudhary R, Khan JA. 2010. Ecology of leopard (Panthera pardus fusca Meyer) in dry tropical forests of Gir National Park and Sanctuary, Gujarat, India. *International Journal of Ecology and Environmental Sciences* 45:241–255.
