# Supplementary material for: Animal movement ecology in India: insights from 2011–2021 and prospective for the future
Source: PeerJ. 2022 Dec 13;10:e14401. doi: 10.7717/peerj.14401 (PMC9756863; doi:10.7717/peerj.14401)
Supplement: Supplemental Information 3 [file peerj-10-14401-s003.docx]

**Identification of new studies via other methods**

**Previous studies**

**Identification of new studies via databases and registers**

Studies included in previous version of review (n = NA)

Reports of studies included in previous version of review (n = NA)

Records identified from*:

Databases (n = **1510**)

Registers (n = )

Records removed *before screening*:

Duplicate records removed (n = )

Records marked as ineligible by automation tools (n = )

Records removed for other reasons (n = )

Records identified from:

Websites (n = )

Organisations (n = **7***)

Citation searching (n = )

Other (n = **6^#^**)

*Wildlife Institute of India

#Experts in the field shared publication

**Identification**

Total studies included in review

(n = **79**)

Reports of total included studies

(n = **3**)

Reports assessed for eligibility

(n = )

Reports sought for retrieval

(n = )

Records screened

(n = )

Records excluded**

(n = )

Reports not retrieved

(n = )

Reports sought for retrieval

(n = )

Reports not retrieved

(n = )

**Screening**

Reports excluded:

Reason 1 (n = )

Reason 2 (n = )

Reason 3 (n = )

etc.

Reports excluded:

Reason 1 (n = )

Reason 2 (n = )

Reason 3 (n = )

etc.

Reports assessed for eligibility

(n = )

New studies included in review

(n = 79)

Reports of new included studies

(n = 3)

**Included**

*Consider, if feasible to do so, reporting the number of records identified from each database or register searched (rather than the total number across all databases/registers).

**If automation tools were used, indicate how many records were excluded by a human and how many were excluded by automation tools.

From: Page MJ, McKenzie JE, Bossuyt PM, Boutron I, Hoffmann TC, Mulrow CD, et al. The PRISMA 2020 statement: an updated guideline for reporting systematic reviews. BMJ 2021;372:n71. doi: 10.1136/bmj.n71. For more information, visit: <http://www.prisma-statement.org/>
